# Classification and phylogenetic analyses of the Arabidopsis and tomato G-type lectin receptor kinases

**DOI:** 10.1186/s12864-018-4606-0

**Published:** 2018-04-06

**Authors:** Marcella A. Teixeira, Alex Rajewski, Jiangman He, Olenka G. Castaneda, Amy Litt, Isgouhi Kaloshian

**Affiliations:** 1 Department of Nematology, University of California, Riverside, California USA; 2 Department of Botany and Plant Sciences, University of California, Riverside, California USA; 3 Chaffey College, Rancho Cucamonga, California USA; 4 Institute for Integrative Genome Biology, University of California, Riverside, California USA

**Keywords:** G-LecRKs, Tomato, *Solanum lycopersicum*, *Arabidopsis thaliana*, Lectin receptor, Receptor kinase, *Aquilegia coerulea*, Columbine, Phylogenetic analysis

## Abstract

**Background:**

Pathogen perception by plants is mediated by plasma membrane-localized immune receptors that have varied extracellular domains. Lectin receptor kinases (LecRKs) are among these receptors and are subdivided into 3 classes, C-type LecRKs (C-LecRKs), L-type LecRKs (L-LecRKs) and G-type LecRKs (G-LecRKs). While C-LecRKs are represented by one or two members in all plant species investigated and have unknown functions, L-LecRKs have been characterized in a few plant species and have been shown to play roles in plant defense against pathogens. Whereas Arabidopsis G-LecRKs have been characterized, this family of LecRKs has not been studied in tomato.

**Results:**

This investigation updates the current characterization of Arabidopsis G-LecRKs and characterizes the tomato G-LecRKs, using LecRKs from the monocot rice and the basal eudicot columbine to establish a basis for comparisons between the two core eudicots. Additionally, revisiting parameters established for Arabidopsis nomenclature for LecRKs is suggested for both Arabidopsis and tomato. Moreover, using phylogenetic analysis, we show the relationship among and between members of G-LecRKs from all three eudicot plant species. Furthermore, investigating presence of motifs in G-LecRKs we identified conserved motifs among members of G-LecRKs in tomato and Arabidopsis, with five present in at least 30 of the 38 Arabidopsis members and in at least 45 of the 73 tomato members.

**Conclusions:**

This work characterized tomato G-LecRKs and added members to the currently characterized Arabidopsis G-LecRKs. Additionally, protein sequence analysis showed an expansion of this family in tomato as compared to Arabidopsis, and the existence of conserved common motifs in the two plant species as well as conserved species-specific motifs.

**Electronic supplementary material:**

The online version of this article (10.1186/s12864-018-4606-0) contains supplementary material, which is available to authorized users.

## Background

In the constant war against pathogens, plants are equipped with a surveillance system that relies on pattern-recognition receptors (PRRs), proteins localized at the plasma membrane with ectodomains, that screen the environment for conserved microbial or pest- and damage-associated signals. In addition to the ectodomain, a subgroup of these PRRs has intracellular kinase domains and are therefore known as receptor kinases (RKs). Plant RKs have undergone a recent expansion, with the *Arabidopsis thaliana* (Arabidopsis) genome encoding more than 600 RKs [1]. According to their ectodomains, RKs can be further classified into specific subgroups, such as leucine-rich repeat RKs (LRR-RKs) and lectin RKs (LecRKs). Receptor kinases are involved in several cellular processes, from adaptation to abiotic stresses to defense responses against pathogens and pests and interactions with microbial symbionts [2–14]. Several RKs and their cognate elicitor pairs have been described, mostly engaging the recognition of bacteria and fungi by plants [14–16].

The best-characterized PRR-elicitor pair is the Arabidopsis LRR-RK FLS2 (FLAGELLIN SENSITIVE2) and the peptide flg22, consisting of a stretch of 22 amino acids of the N-terminal bacterial flagellin [17]. In addition to Arabidopsis, FLS2 orthologs have been identified in several plant species including tomato (*Solanum lycopersicum*), grapevine (*Vitis vinifera*), rice (*Oryza sativa*) and citrus (*Citrus paradisi*, *C. reticulata* and *Fortunella margarita*) [18–21]. Interestingly in tomato, a flagellin-derived peptide distinct from flg22, flgII-28, is perceived by the LRR-RK FLS3, and similar to FLS2, its perception and downstream signaling requires a second LRR-RK, BAK1 (BRASSINOSTEROID INSENSITIVE 1-ASSOCIATED KINASE 1) [22, 23]. Other receptor-ligand pairs include chitin perception by the lysin-motif RK (LysM-RKs) LYK5 and xylanase perception by the LRR-RK EIX2 [24, 25]. Although a co-receptor has not been characterized for xylanase perception, chitin perception requires participation of the LysM-RK CERK1 (CHITIN ELICITOR RECEPTOR KINASE 1) [26]. Interestingly, chitin perception in rice is mediated by the LysM-receptor like protein CEBiP (CHITIN ELICITOR BINDING PROTEIN), which lacks a kinase domain and relies on its co-receptor CERK1 for kinase signaling of chitin perception [27].

**Fig. 1:**
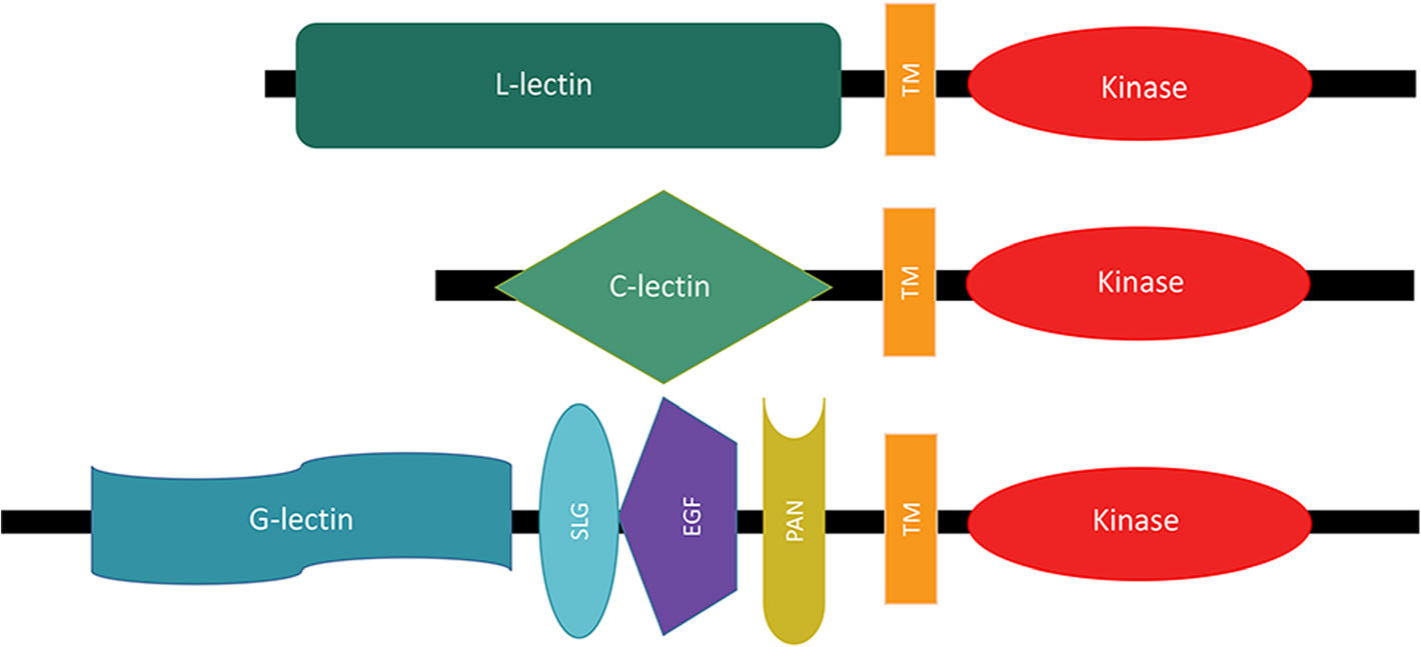
Domains of lectin receptor kinases (LecRKs). G-lectin, C-lectin and L-lectin are the motifs in the ectodomains of G-type, C-type, and L-type LecRKs, respectively

The LecRKs are a second type of receptors known for their role in binding various carbohydrates [9]. Based on their ectodomains, LecRKs can be classified into C-type, L-type or G-type (Fig. 1). The C-type (calcium-dependent) LecRKs (C-LecRK) contain the C-type motif that is commonly found in several proteins from mammals, and these proteins have been shown to have a role in innate immunity [10, 28, 29]. Interestingly, in plants this LecRK group is represented by only a single gene in Arabidopsis, rice, and tomato, and two genes in wheat (*Triticum aestivum*) [28, 30, 31].

The L-type (legume-like) LecRKs (L-LecRKs) consist of large number of family members and have well-characterized roles in plant defense. Investigations in Arabidopsis, rice, tomato, *Nicotiana benthamiana* and wheat revealed 45, 72, 22, 37, 84 members of this family, respectively [28, 30, 31]. Several reports link genes of this family to defense against pathogens; for example, AtLecRK-I.9 against the bacterial pathogen *Pseudomonas syringae* pv. *tomato (Pst)* [32], AtLecRK-IX.I and LecRK-IX.2 against the pathogenic oomycetes *Phytophthora brassicae* and *P. capsici* [33], AtLecRK-I.9 against *P. infestans* [34], and AtLecRK-VI.2 against the pathogenic bacteria *P. syringae* and *Pectobacterium carotovorum* [13, 35]. Additionally, L-LecRKs have been implicated in perception of the danger molecule, the extracellular ATP, by the AtLecRK-I.9 [36, 37].

The G-type LecRKs (G-LecRKs) are proteins with an ectodomain that resembles the *Galanthus nivalis* agglutinin (GNA) mannose-binding motif [10, 38]. Since the discovery of the first G-LecRK, GNA, in 1987, this group has been renamed and refined several times [39]. The specificity of GNA for binding mannose differentiated it from the mannose/glucose specificity of L-LecRKs and led other proteins of this group to be generically called alpha-D-mannose-specific lectins [39, 40]. Soon after the discovery of GNA, several studies targeting other members of Amaryllidaceae, along with Orchidaceae and Alliaceae discovered more mannose-specific lectins [41, 42]. Based on the apparently narrow taxonomic occurrence of the proteins at the time, the group was then renamed monocot mannose-binding lectins. Early amino acid sequence-based analyses also began to suggest that these proteins might be encoded by a large multigene family [43]. Subsequently, similar proteins were discovered in both liverwort (*Marchantia polymorpha*) and yew (*Taxus media*) necessitating another renaming to the more general GNA-related lectins [44, 45]. Because GNA was first isolated from *Galanthus nivalis* bulbs and other G-LecRKs were present in high amounts in bulbs of other species, this group has also been called Bulb- or B-type lectins. This name has been widely adopted because it is used to identify this domain in both the PROSITE and Pfam databases (PS50927 and PF01453, respectively). Despite the widespread use of the “B-type” descriptor in databases and genome annotations, GNA-related or G-type lectin is currently the preferred term for these proteins.

Previous investigations identified 32 members of G-LecRKs in Arabidopsis, 100 in rice and 177 in wheat [28, 31]. The best-known members of this group are the S-locus (S-locus glycoprotein/SLG containing) RKs, known for their role in self-incompatibility in flowering plants [46, 47]. Besides the G-type lectin and the kinase domains, G-LecRKs can have additional domains such as a cysteine-rich domain (Fig. 1), known as the epidermal growth factor (EGF) domain, which is thought to play a role in disulfide bond formation [48]. Additionally, family members may contain the plasminogen-apple-nematode (PAN) motif, which likely has a role in protein-protein or protein-carbohydrate interactions (Fig. 1) [49].

Typically, members of large families do not have consistent nomenclature, as frequently not all members are identified at the same time, and gains and losses in different species make identification of orthologous genes and gene groups difficult. While the Arabidopsis L-LecRK family members have a clear systematic nomenclature based on chromosome location and amino acid and nucleotide identity [34], currently members of the G-LecRKs do not have such nomenclature. Similarly, although L-LecRKs have been characterized in different plant species [28, 30, 31], tomato G-LecRKs have not been described to date. Given the role of these proteins in defense, an understanding of their diversity in tomato is critical to improving crop resistance to diseases and pests.

For this work, we searched the genomes of Arabidopsis and tomato to identify and characterize G-LecRKs, and performed phylogenetic analyses on the aligned sequences, using C-LecRKs and L-LecRKs as outgroups. To draw inferences regarding expansion vs loss within gene clades, we included G-LecRKs from the monocot rice and the basal eudicot columbine (*Aquilegia coerulea*), which diverged prior to the origin of the core eudicot clade that includes both Arabidopsis and tomato and thus serves to polarize the evolutionary trends. The analyses allowed identification of incorrect gene annotations in genome databases, and evaluation of sequence similarity between G-LecRKs and identification of instances of gene clade expansion or gene loss in Arabidopsis and tomato. Based on this investigation, we suggest a nomenclature for members of this gene family from both Arabidopsis and tomato.

## Methods

### Database searches, protein domain and genome organization

To identify Arabidopsis (*Aarabidopsis thaliana*) G-LecRKs a first search was performed using the lectin domain of At1g61550 as the query followed by the lectin domain of At1g61400, At2g19130, At4g21390 and At5g60900 for a second search in The Arabidopsis Information Resource (TAIR) (http://arabidopsis.org) website. Results with e-value < 0.1 were considered G-LecRKs candidates. The localization of G-LecRKs on the Arabidopsis genome was visualized using the chromosomal map tool from TAIR (http://arabidopsis.org/jsp/ChromosomeMap/tool.jsp). Arabidopsis predicted kinase domain sequences were aligned using ClustalW and the alignment was manually checked to identify the kinase subdomains using *At*LecRK-VI.2 as a reference [13, 30].

To identify tomato (*Solanum lycopersicum*) G-LecRKs, the At1g61550 lectin domain was used as the query in the Sol Genomics Network (SGN) (https://solgenomics.net) and at the National Center for Biotechnology Information (NCBI) (https://www.ncbi.nlm.nih.gov/) websites. Results with e-value < 0.1 were considered G-LecRKs candidates. After the initial search using the At1g61550 G-lectin domain, a second search was performed in NCBI and both searches were cross analyzed to compile a list of all possible G-LecRK candidates. Tomato G-LecRKs sequences were mapped according to Shearer et al. [50]. To map tomato sequences onto chromosomes, gene position information was acquired from Phytozyme (https://phytozome.jgi.doe.gov/pz/portal.html) and JBrowse at SGN. Mapping of G-LecRKs onto tomato chromosomes was performed manually using NCBI Map Viewer (https://www.ncbi.nlm.nih.gov/genome/gdv/). Tomato predicted kinase domain sequences were aligned with the kinase domain of Solyc03g006720 using ClustalW and the kinase subdomains were manually checked to identify the kinase subdomains.

To identify columbine (*Aquilegia coerulea*) G-LecRKs, the same Arabidopsis gene (At1g61550) was used in a BLASTp search of the genome sequence hosted by Phytozome. Three hits from this search were then chosen for another round of searching with BLASTp. Only BLAST hits with an e-value < 0.1 were retained. A search was also conducted using the keyword “lectin”. The resulting sequences were passed to IPRscan on the University of California at Riverside (UCR) High-Performance Computing Center (HPCC) cluster for protein domain identification using Pfam and Prosite [51].

For G-LecRK identification in rice (*Oryza sativa*), in addition to the 87 proteins listed in Vaid et al. [28], protein sequences annotated with the Pfam code PF01453 were retrieved from the Rice Genome Annotation Project (http://rice.plantbiology.msu.edu/). All sequences were passed to IPRscan using the same parameters as for columbine.

### Phylogenetic analysis

Full-length protein sequences were downloaded as above from TAIR (Arabidopsis), Sol Genomics Network (tomato), Phytozome (columbine), or the Rice Genome Annotation Project (rice) and aligned using MUSCLE on the UCR HPCC cluster [52]. Eight outgroup protein sequences representing one C-LecRK and L-LecRK each from Arabidopsis, tomato, rice and columbine were included in the alignments. These sequences were from the single Arabidopsis C-LecRK gene, At1g52310, and its top BLASTp hits from tomato (Solyc02g068370.2), rice (Os01g0104000.1), and columbine (Aqcoe2G393700); and an Arabidopsis L-LecRK (At1g52310) and its top BLASTp hits from tomato (Solyc02g068370.2), rice (Os04g0531500.1) and columbine (Aqcoe2G393700). The initial sequence alignment was generated using data from Arabidopsis, tomato, columbine, and rice. Subsequently, a three-species alignment was generated using only sequences from Arabidopsis, tomato, and columbine.

The JTT model of protein sequence evolution was determined to best fit the data based on Akaike Information Criteria using the web server version of SMS, and phylogenetic trees were subsequently constructed from both the four-species and three-species amino acid alignments using this evolutionary model in RAxML v8 on the UCR HPCC cluster with 1000 bootstraps [53–55]. A collapsed tree was also constructed from the three- and four- species trees in TreeGraph 2 by collapsing any node with < 70% bootstrap support into a polytomy [56].

### Domain and motif identification

Protein domains were investigated using multiple online programs including, InterPro (https://www.ebi.ac.uk/interpro/) and TMHMMM (http://www.cbs.dtu.dk/services/TMHMM/). Investigation of conserved motifs in the ectodomains of Arabidopsis and tomato G-LecRKs was performed using the default settings at MEME (Multiple EM for Motif Elicitation) Suite 4.11.2 (http://meme-suite.org/tools/meme) [57].

### Subcellular localization prediction

Multiple protein subcellular localization tools were used to localize the Arabidopsis and tomato G-LecRKs. Arabidopsis gene identifiers were used to query “The SUBcellular localization database for Arabidopsis proteins”, SUBA3 (http://suba3.plantenergy.uwa.edu.au/) [58, 59]. Additionally, amino acid sequences of both Arabidopsis and tomato G-LecRKs were analyzed using TargetP 1.1 Server (http://www.cbs.dtu.dk/services/TargetP/) and “subCELlular LOcalization predictor” CELLO v.2.5 (http://cello.life.nctu.edu.tw/) [60, 61].

## Results

### Annotation of Arabidopsis G-LecRKs

To identify the Arabidopsis G-LecRKs, a BLASTp analysis [62] was performed at the TAIR website using the region comprising the predicted G-type lectin domain [63], amino acids 24-170 from At1g61550. The search resulted in 44 sequences. From these sequences, four (At1g61400, At2g19130, At4g21390 and At5g60900) were chosen for use as new queries to fish out additional candidates. These analyses resulted in a total of 49 proteins with a G-lectin domain (Table 1). The majority of these proteins had SLG (75%) and PAN (82%) domains but only 10 had an EGF domain. Of these 49 sequences, 38 proteins had also kinase domains and were considered for further analyses (Table 1).

**Table 1:** Domains of Arabidopsis genes encoding G-Lectins

	Locus	^a^SLG	EGF	PAN	TM	Kinase
1	AT1G11340	^b^x	x	x	x	x
2	AT1G11410	x	x	x	x	x
3	AT1G61360	x	x	x	x	x
4	AT1G61380	x	x	x	x	x
5	AT1G61390	x	x	x	x	x
6	AT1G61550	x	x	x	x	x
7	AT1G61610	x	x	x	x	x
8	AT2G19130	x	x	x	x	x
9	AT4G27290	x	x	x	x	x
10	AT4G03230	x	x	x	x	x
11	AT1G11280	x	–	x	x	x
12	AT1G11300	x	–	x	x	x
13	AT1G11305	x	–	x	x	x
14	AT1G11330	x	–	x	x	x
15	AT1G11350	x	–	x	x	x
16	AT1G61370	x	–	x	x	x
17	AT1G61400	x	–	x	x	x
18	AT1G61420	x	–	x	x	x
19	AT1G61430	x	–	x	x	x
20	AT1G61440	x	–	x	x	x
21	AT1G61480	x	–	x	x	x
22	AT1G61490	x	–	x	x	x
23	AT1G61500	x	–	x	x	x
24	AT1G65790	x	–	x	x	x
25	AT1G65800	x	–	x	x	x
26	AT2G41890	x	–	x	x	x
27	AT4G11900	x	–	x	x	x
28	AT4G21380	x	–	x	x	x
29	AT4G21390	x	–	x	x	x
30	AT4G27300	x	–	x	x	x
31	AT1G34300	x	–	–	x	x
32	AT4G00340	x	–	–	x	x
33	AT5G24080	x	–	–	x	x^c^
34	AT3G16030	–	–	x	x	x
35	AT4G32300	–	–	–	x	x
36	AT5G35370	–	–	–	x	x
37	AT1G67520	–	–	x	–	x^c^
38	AT5G60900	–	–	x	–	x
39	AT5G03700.1	x	–	x	x	–
40	AT1G78830	–	–	x	x	–
41	AT3G12000	x	–	x	x	–
42	AT3G51710.1	x	–	x	x	–
43	AT1G16905	–	–	–	x	–
44	AT5G18470	–	–	–	x	–
45	AT1G78820	–	–	x	–	–
46	AT1G78850	–	–	x	–	–
47	AT1G78860	–	–	x	–	–
48	AT2G01780	–	–	–	–	–
49	AT5G39370	–	–	–	–	–

^a^*SLG* S-locus glycoprotein, *EGF* epidermal growth factor, *PAN* plasminogen apple nematode, *TM* transmembrane

^b^“x” denotes presence and “-” denotes absence of a domain

^c^Incomplete kinase domain

Previous characterization of Arabidopsis G-LecRKs included 31 sequences [28], all also identified in our search. This same study also identified a protein, At1g61460, that was not detected in our search. To confirm the identity of this protein, it was used in domain search using InterPro. Domain predictions showed that At1g61460 has SLG, PAN, transmembrane (TM) and kinase domains, but not a lectin domain. Because of the absence of the lectin domain, this protein was not considered a G-LecRK and was not used in further analyses.

### Annotation of tomato G-LecRKs

Using the same strategy used to retrieve the Arabidopsis G-LecRKs, the tomato genome was queried for G-type lectin homologs using the lectin domain of At1g61550. Two databases, SGN and NCBI, were searched. The search against SGN resulted in 21 sequences. The search against NCBI resulted in numerous hits, including a number of different isoforms of variable lengths of the same protein. The combined results from these two searches yielded 88 distinct sequences with a G-type lectin domain (Table 2). To assure a comprehensive search, three random tomato G-type lectins were chosen to query the tomato genome again using their predicted G-lectin domain. This resulted in two additional candidates, Solyc07g053220 and Solyc05g008310.

**Table 2:** Domains of tomato genes encoding G-Lectins

	Locus	^a^SLG	EGF	PAN	TM	Kinase
1	Solyc02g079640	^b^x	x	x	x	x
2	Solyc04g008400.A	x	x	x	x	x
3	Solyc04g008400.B	x	x	x	x	x
4	Solyc04g058110	x	x	x	x	x
5	Solyc07g063770	x	x	x	x	x
6	Solyc10g006710	x	x	x	x	x
7	Solyc11g005630	x	x	x	x	x
8	Solyc01g094830	x	–	x	x	x
9	Solyc02g030300	x	–	x	x	x
10	Solyc02g079530	x	–	x	x	x
11	Solyc02g079540	x	–	x	x	x
12	Solyc02g079550	x	–	x	x	x
13	Solyc02g079570	x	–	x	x	x
14	Solyc02g079590	x	–	x	x	x
15	Solyc02g079620	x	–	x	x	x
16	Solyc02g079630	x	–	x	x	x
17	Solyc02g079710	x	–	x	x	x
18	Solyc03g006720	x	–	x	x	x
19	Solyc03g006730.A	x	–	x	x	x
20	Solyc03g006730.B	x	–	x	x	x
21	Solyc03g006770	x	–	x	x	x
22	Solyc03g006780	x	–	x	x	x
23	Solyc03g063650	x	–	x	x	x
24	Solyc04g008370	x	–	x	x	x
25	Solyc04g077270	x	–	x	x	x
26	Solyc04g077280	x	–	x	x	x
27	Solyc04g077300	x	–	x	x	x
28	Solyc04g077340	x	–	x	x	x
29	Solyc04g077360	x	–	x	x	x
30	Solyc04g077370	x	–	x	x	x
31	Solyc04g077390	x	–	x	x	x
32	Solyc04g078410	x	–	x	x	x
33	Solyc05g008310	x	–	x	x	x
34	Solyc07g053080	x	–	x	x	x
35	Solyc07g053120	x	–	x	x	x
36	Solyc07g053130	x	–	x	x	x
37	Solyc07g053220	x	–	x	x	x
38	Solyc07g063700	x	–	x	x	x
39	Solyc07g063710	x	–	x	x	x
40	Solyc07g063720	x	–	x	x	x
41	Solyc07g063730	x	–	x	x	x
42	Solyc07g063750	x	–	x	x	x
43	Solyc07g063780	x	–	x	x	x
44	Solyc07g063800	x	–	x	x	x
45	Solyc09g011330	x	–	x	x	x
46	Solyc10g005440	x	–	x	x	x
47	Solyc10g006720	x	–	x	x	x
48	Solyc12g005290	x	–	x	x	x
49	Solyc01g006520	x	–	–	x	x
50	Solyc03g005130	x	–	–	x	x
51	Solyc03g007790	x	–	–	x	x
52	Solyc03g078360	x	–	–	x	x
53	Solyc03g078370	x	–	–	x	x
54	Solyc06g036470	x	–	–	x	x
55	Solyc09g075910	x	–	–	x	x
56	Solyc09g075920	x	–	–	x	x
57	Solyc11g013880	x	–	–	x	x
58	Solyc02g072070	–	–	x	x	x
59	Solyc03g120110	–	–	x	x	x
60	Solyc08g076050	–	–	x	x	x
61	Solyc08g076060	–	–	x	x	x
62	Solyc12g006840	–	–	x	x	x
63	Solyc01g006530	–	–	–	x	x
64	Solyc04g015460	–	–	–	x	x
65	Solyc04g077380	–	–	–	x	x
66	Solyc07g055650	–	–	–	x	x
67	Solyc08g059730	–	–	–	x	x
68	Solyc07g063820	x	–	x	–	x
69	Solyc07g063810	–	–	x	–	x
70	Solyc07g055630	–	–	–	–	x
71	Solyc07g055640.A	–	–	–	–	x
72	Solyc07g055640.B	–	–	–	–	x
73	Solyc08g076070	–	–	–	–	x
74	Solyc04g077310	x	–	–	x	–
75	Solyc07g053090	x	–	x	x	–
76	Solyc09g009150	x	–	x	x	–
77	Solyc09g018490	x	–	–	x	–
78	Solyc02g076830	x	–	x	–	–
79	Solyc10g006690	x	–	x	–	–
80	Solyc04g077320	x	–	–	–	–
81	Solyc07g009440	x	–	–	–	–
82	Solyc07g055690	x	–	–	–	–
83	Solyc01g014510	–	–	–	–	–
84	Solyc01g014540	–	–	–	–	–
85	Solyc01g014560	–	–	–	–	–
86	Solyc01g014640	–	–	–	–	–
87	Solyc01g014700	–	–	–	–	–
88	Solyc02g030340	–	–	–	–	–
89	Solyc02g030380	–	–	–	–	–
90	Solyc02g078730	–	–	–	–	–
91	Solyc07g009410	–	–	–	–	–
92	Solyc07g062480	–	–	–	–	–
93	Solyc07g062490	–	–	–	–	–

^a^*SLG* S-locus glycoprotein, *EGF* epidermal growth factor, *PAN* plasminogen apple nematode, *TM* transmembrane

^b^“x” denotes presence and “–” denotes absence of a domain

Three of the identified G-type lectin-containing sequences were misannotated. Solyc03g006730, Solyc04g008400, and Solyc07g055640 each contained two G-LecRKs in tandem and were therefore each split into two (Solyc03g006730.A and Solyc03g006730.B; Solyc04g008400.A and Solyc04g008400.B; and Solyc07g055640.A and Solyc07g055640.B) (Table 2). Thus, a total of 93 tomato sequences were identified with G-type lectin domains. The majority (72%) of these tomato sequences had an SLG domain and about half (63.5%) had a PAN domain. However, similar to Arabidopsis, the great majority lacked the EGF domain, with only seven proteins containing this domain (Table 2). Of the 93 tomato sequences, 73 proteins had both G-type lectin and kinase domains and were considered G-LecRKs for further analyses.

### Annotation of columbine G-LecRKs

For columbine G-LecRK identification, the same Arabidopsis G-LecRK sequence, At1g61550, was used as a query sequence for a BLASTp search of the genome sequence. Three hits from this search were then chosen for another round of searching with BLASTp. Taking advantage of the functional genome annotations available, a keyword search was also conducted of the columbine genome using the keyword “lectin”. After merging duplicates, this yielded 166 unique protein sequences. Of these, two could not be annotated at all, while 43 others lacked a G-type lectin domain, kinase domain, or both (Additional file 1). Of the 121 columbine sequences, 59 proteins had both G-type lectin and kinase domains and were used in the phylogenetic analysis.

### Annotation of rice G-LecRKs

For G-LecRK identification in rice, the 87 previously published proteins were all included [28]. In addition, protein sequences annotated with the Pfam code PF01453 (B-type lectin, synonymous with G-type lectin) were retrieved from the genome sequence. This generated 143 sequences, including the 87 previously reported, but also including different isoforms of some proteins. In the case of isoforms, only the longest was retained. Additionally, LOC_Os09g37840.1 appeared to be a misannotation containing three G-LecRKs in tandem. This locus was split into three sequences (LOC_Os09g37840.1.A,. B, and. C). This resulted in 145 protein sequences with G-type lectin domain (Additional file 2). Of these 145, 122 rice sequences had also a kinase domain and were used in the phylogenetic analysis.

### Phylogenetic analysis

Alignment of the 38 Arabidopsis, 73 tomato, 59 columbine, and 122 rice putative G-LecRK proteins was used to construct a phylogenetic tree with 1000 bootstrap replicates using RaxML [55]. Orthologous copies of a single C- and L-LecRK from Arabidopsis, tomato, rice, and columbine were used as outgroups to root the tree. In this four-species analysis, the C-LecRKs form a sister-clade to a large clade that includes all the other genes, including the L-LecRKs (Additional file 3). The clade containing all of the putative G-LecRKs and the intended outgroup L-LecRKs is further divided into two large clades, although with weak support. The L-LecRKs are included as the first branch in one of these two clades, suggesting that in a phylogenetic context, they should be considered G-LecRKs. In general, support in this tree for deep nodes is weak, with much stronger support towards the tips, suggesting rapid diversification of this gene group, and making interpretation of clade relationships difficult. In addition, the large number of rice genes largely cluster into clades that are nearly or entirely rice-specific (Additional file 3). This indicates extensive expansion of the G-LecRKs independently in rice relative to the eudicots, and makes interpretation of the relationships between Arabidopsis and tomato genes more difficult. For that reason, we performed a second phylogenetic analysis including only Arabidopsis, tomato, and columbine.

The results of this three-species analysis (Additional file 4) mirror those of the four-species analysis, with the C-LecRK outgroup as sister to a large clade that includes both the L- and G-LecRKs. As in the first analysis, this clade is further subdivided into two major clades, one of which (Clade A) includes the L-LecRKs, again suggesting they are not a group distinct from the G-LecRKs. Also consistent with the first analysis, support for deep nodes is weak. For instance, the first branch of clade A (Additional file 4) consists of the three L-LecRK genes that were intended to serve as outgroup with the C-LecRKs. The next branch, with 30% support, includes only one gene each from Arabidopsis and tomato, and the following branch, with 24% support, includes 8 genes from columbine only. This implies that a columbine gene has been lost from the first clade, and that Arabidopsis and tomato genes have been lost from the second. With such low support, however, it is not possible to rule out a topology in which all these genes are members of a single clade, with one Arabidopsis, one tomato, and 8 columbine genes, indicating diversification in columbine rather than multiple losses.

**Fig. 2:**
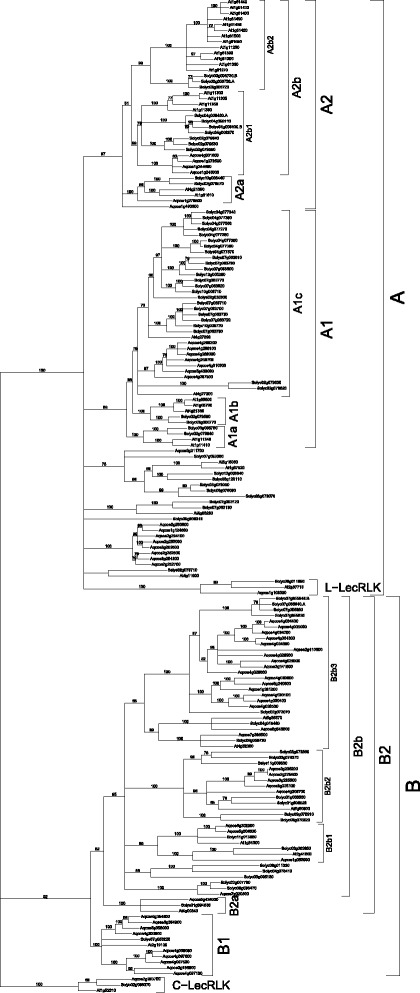
Maximum likelihood tree of amino acid sequences of G-LecRK sequences from Arabidopsis, tomato, and columbine, with L-LecRK and C-LecRK sequences from each species as outgroup (but see text regarding L-LecRK sequence placement). Bootstrap support from 1000 replicates is shown above nodes. Nodes with < 70% bootstrap support were collapsed into polytomies. Brackets on the right indicate hierarchical clade names (or outgroups) as defined in the text

Because of the weak support at many key nodes, for analysis of clade relationships and membership, we used a tree in which all nodes with less than 70% support were collapsed into polytomies (Fig. 2). Although the resulting topology includes fewer resolved relationships, those represented are more robustly supported in the data, providing a stronger basis for exploring expansions and losses in Arabidopsis and tomato. The two large clades remain, although their relationship to the outgroup is unresolved. This suggests an early duplication, prior to the divergence of monocots, producing two independently diverging clades of G-LecRKs. Available data do not show a clear distinction between these groups in function or expression; members of both groups show response (functional or regulatory) to various biotic and abiotic stresses [47, 64–69]. Several members of clade A (Fig. 2) are implicated in growth and development processes, which have so far not been reported for clade B members, however relatively few clade B members have been characterized at any level [70, 71].

A glance at the phylogenetic tree shows that within clades, there has been dramatically differential expansion of specific G-LecRK lineages in specific species. For the most part, Arabidopsis, tomato, and columbine genes form distinct species-specific groups within larger clades, suggesting that there have been multiple duplications leading to a large number of gene clades, but that after these duplications, there has been separate diversification of gene groups within each species. Expression and functional date are currently available only for Arabidopsis genes, and the diversity of functions, even within a single clade, suggests that this is a rapidly evolving group [66, 72–80]. This makes prediction of function in tomato difficult in most cases.

Clade A1, with 88% support, includes a total of 7 columbine, 7 Arabidopsis, and 28 tomato sequences. This clade is further subdivided into two successive lineages with multiple Arabidopsis and tomato genes but no columbine gene, suggesting loss of columbine genes and additional duplications separately within both Arabidopsis and tomato. The first clade (A1a) includes the Arabidopsis gene At1g11340.1, the expression of which has been shown to be suppressed by mevalonic acid [73]. The second (A1b) includes the S-locus/ARK3 gene At4g21380.1, involved in self incompatibility, which is sister to the sister-gene pair At1g65790.1, upregulated by Fusarium, salt, and flg22, and At1g65800.1 (SD1-6/ARK2), involved in lateral root formation [78, 79, 81, 82]. Clade A1a is sister to a large clade (A1c) with poor internal resolution that includes 7 columbine, 2 Arabidopsis, and 24 tomato genes, indicating dramatic relative expansion within tomato relative to the other two species. Of the two Arabidopsis genes found in this clade, At4g27300.1 is upregulated by osmotic stress and trehalose, and downregulated by sucrose, whereas At4g27290.1 is induced by cold [72, 83]. The wide variation in reported functions for these Arabidopsis genes, and the lack of data for the other species, makes it difficult to generalize regarding the function of genes of individual clades and therefore to extrapolate and predict the functions of the tomato genes.

Clade A includes one other large clade with 97% support (A2) that is further subdivided into two clades with one columbine gene (Aqcoe1g490600) that does not fall into either. Clade A2a, with 100% support, includes 5 genes: one columbine and two each Arabidopsis and tomato. Because the two tomato genes form a sister-pair, as do the two Arabidopsis genes, this indicates independent duplications in those two species. At1g61610.1 has been shown to be upregulated by flg22, bacterial lipopolysaccharide (LPS), and *Pst* strain DC3000, suggesting a role in defense against bacterial pathogens [69, 84]. Its paralog At4g21390.1 is also implicated in defense and is upregulated in the presence of fungal elicitors [72]. Although it is clear the function of these genes is highly labile, overall it suggests a role in defense for this clade.

Clade A2b, with 81% support, includes two subclades, both with 99% support. Both clades include sequences from both Arabidopsis and tomato, but only one (A2b1) also includes columbine, indicating a duplication that produced the two clades but loss of the columbine gene from one of the clades (A2b2). Both of these sister-clades have experienced expansion in both tomato and Arabidopsis, but A2b1 has a somewhat greater number in tomato (7 vs 4 in Arabidopsis), whereas A2b2 has the reverse, with substantially more in Arabidopsis (13 vs 3 in tomato). The presence of distinct Arabidopsis and tomato genes again suggests, in these two clades, independent expansion in the two species. Clade A2b1 includes Arabidopsis EGM1 (ENHANCED GROWTH ON MANNITOL1; At1g11300) and EGM2 (AT1g11305), paralogs implicated in plant shoot growth and mannitol stress, as well as CBRLK1 (CALMODULIN-BINDING RECEPTOR-LIKE CYTOPLASMIC KINASE1; At1g11350), a negative regulator of immunity against *Pst*, and At1g1130.2, which likely also plays a role in bacterial immunity [65, 70, 72]. The topology of the clade suggests that the role for shoot growth is derived. Expression of the Arabidopsis genes in clade A2b2 have been shown to respond to a wide variety of factors and, as with other clades, to play roles in plant development and defense responses [71, 75, 76, 84, 85]. Among these is the LPS receptor LORE (LIPOPOLYSACCHARIDE-SPECIFIC REDUCED ELICITATION/ SD1-29; At1g61380), known to mediate LPS sensitivity in Brassicaceae [66]. This LPS sensitivity does not appear to be in solanaceae [66].

Patterns of diversification are more varied and complex in clade B, and it includes only 7 genes from Arabidopsis, along with 23 from tomato and 37 from columbine. This clade is divided into two subclades, one of which (B1, 100% support) shows diversification in columbine, with 9 genes compared to one each in Arabidopsis and tomato. Although this Arabidopsis gene (At2g19130) has not been characterized, its ortholog in rice (*OsSIK2*; Os07g0186200) is implicated in salt and drought response [86]. The other clade, B2 (82% support), is further subdivided into one small clade that includes only one gene from each species, and a large clade with multiple subclades. This small clade B2a (100% support), one of the very few clades in the tree that has only one gene per species, has no evidence of diversification in any of the species. The Arabidopsis gene (At4g00340.1) has not been characterized, but its ortholog in strawberry (*M2F10*) is upregulated in response to infection by the fungus *Colletotrichum acutatum* [87]. Given that many other G-LecRKs respond to fungal pathogens, it is difficult to hypothesize why this particular clade has not undergone the type of expansion seen in nearly every other clade.

The sister clade to B2a (B2b, with 95% support) is subdivided into 5 clades that are unresolved relative to each other. One of these (B2b1) also shows relatively little expansion: it consists of two sister-clades, each of which has genes from all three species. This indicates a duplication before the diversification of the eudicots, but the only further expansion is a single columbine duplication. The two Arabidopsis genes in clade B2b1 respond to abiotic stress: At2g41890.1 is downregulated in response to gravity, and At1g34300.1, which responds to water loss and decreased dry weight [72, 74]. This suggests a role in abiotic stress response for the tomato and columbine genes as well.

Clade B2b contains two larger subclades that show greater diversification. Clade B2b2 (100% support) has one Arabidopsis member, At5g60900.1, which is implicated in both biotic (upregulated by *Fusarium* and LPS) and abiotic (downregulated by cold) responses [72, 78, 88]. Although resolution within this clade is poor, the nested position of the Arabidopsis gene suggests that the low number of genes from this species may be the result of loss. However, further resolution could show instead independent diversification in tomato and columbine. Clade B2b3 (99% support) is subdivided into two sister clades, one of which consists entirely of tomato and columbine genes, strongly suggesting loss in Arabidopsis. The other subclade consists of two sister-clades each of which has one gene from each species, another example of a single duplication followed by no additional diversification in any species. One of the Arabidopsis genes, *SD2-5* (At4g32300.1), is not characterized, but the rice homolog, *Pi-d2*, confers resistance to the fungal pathogen *Magnaporthe grisea* [89]. The other Arabidopsis gene, At5g35370.1, is downregulated in response to nematode infection and is important in salt and drought tolerance, thereby showing roles in both biotic and abiotic responses [90, 91].

### Kinase domain analysis of Arabidopsis G-LecRKs

The presence of the 11 known kinase subdomains and sites essential for the catalytic activity [51] of the G-LecRKs was investigated by aligning the amino acid sequences of the kinase domains to the LecRK-VI.2 kinase domain [13]. The alignment revealed overall conservation of the ATP binding (consensus motif G-x-G-x-x-G-x-V) and the catalytic sites (consensus motif H-R-D-L-K-x-x-N), with a few substitutions in the other kinase subdomains (Additional file 5) [92]. Two of the G-LecRK sequences, At1g67520 and At5g24080, revealed incomplete kinase domains, lacking four (VIII – XI) and seven (I-VII) of the 11 kinase subdomains, respectively. Additionally, At2g41890 has several mutations and deletions notably, in the ATP binding site, in subdomain I, as well as a single amino acid change in its catalytic loop in subdomain VI (Additional file 5) [92]. The essential arginine and aspartic acid residues are substituted for glycine and asparagine, respectively. Therefore, At1g67520, At5g24080, and At2g41890 are likely inactive kinases.

### Nomenclature for the Arabidopsis G-LecRKs

**Fig. 3:**
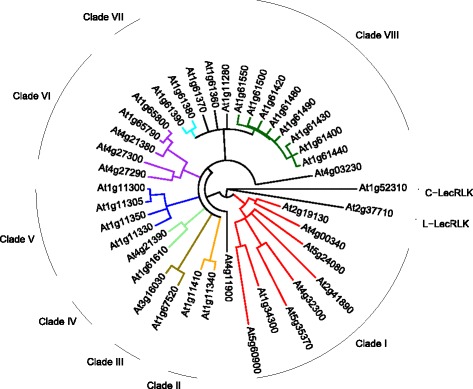
Maximum likelihood tree of amino acid sequences of G-LecRK sequences from Arabidopsis, with L-LecRK and C-LecRK sequences as outgroups. Labeled lines on the outside of the tree represent clade names as defined in the text, and clades are colored to match. Nodes with < 70% bootstrap support were collapsed into polytomies. (Bootstrap percentages not shown)

The L-LecRKs were previously classified and a nomenclature was established based on the amino acid and nucleotide sequences of the 45 members of the family [34]. In that system, clades, defined as groups of genes with at least 50% identity among homologs at both the nucleotide and amino acid level, were designated by Roman numerals [34]. Following a similar approach, but basing clade membership on the results of our phylogenetic analysis rather than percent sequence identity, we classified the 38 members of the Arabidopsis G-LecRK group into eight clades (I-VIII) (Fig. 3, Table 3, Additional file 6). Within each clade, genes are numbered progressively, across chromosomes and clades as in Bouwmeester et al. [34]; therefore, gene #1 in each clade would be the gene on the lowest numbered chromosome with the lowest numerical location (for clade I, At1G34300, designated G-LecRK-I.1) and the final gene would be the one on the highest numbered chromosome with the highest numerical location (for clade I, At5G60900, designated G-LecRK-I.8). The two largest identified clades (clades I and VIII) contain eight members, followed by clades with five and four members (VI and V, respectively) and four clades with two members each (Fig. 3, Table 3). Five genes were not placed in any clade, behaving as singletons. Chromosomal location was not predictive of clade membership, with genes from chromosome 1 being found in all eight clades; in addition, three of the singleton genes are located on chromosome 1 where the vast majority of G-LecRKs are localized.

**Table 3:** Classification and proposed nomenclature of the Arabidopsis G-LecRKs

Clade name	Gene name	Locus
G-LecRK-I	*AtG-LecRK-I.1*	AT1G34300
	*AtG-LecRK-I.2*	AT2G19130
	*AtG-LecRK-I.3*	AT2G41890
	*AtG-LecRK-I.4*	AT4G00340
	*AtG-LecRK-I.5*	AT4G32300
	*AtG-LecRK-I.6*	AT5G24080
	*AtG-LecRK-I.7*	AT5G35370
	*AtG-LecRK-I.8*	AT5G60900
G-LecRK-II	*AtG-LecRK-II.1*	AT1G11340
	*AtG-LecRK-II.2*	AT1G11410
G-LecRK-III	*AtG-LecRK-III.1*	AT1G67520
	*AtG-LecRK-III.2*	AT3G16030
G-LecRK-IV	*AtG-LecRK-IV.2*	AT1G61610
	*AtG-LecRK-IV.2*	AT4G21390
G-LecRK-V	*AtG-LecRK-V.1*	AT1G11300
	*AtG-LecRK-V.2*	AT1G11305
	*AtG-LecRK-V.3*	AT1G11330
	*AtG-LecRK-V.4*	AT1G11350
G-LecRK-VI	*AtG-LecRK-VI.1*	AT1G65790
	*AtG-LecRK-VI.2*	AT1G65800
	*AtG-LecRK-VI.3*	AT4G21380
	*AtG-LecRK-VI.4*	AT4G27290
	*AtG-LecRK-VI.5*	AT4G27300
G-LecRK-VII	*AtG-LecRK-VII.1*	AT1G61380
	*AtG-LecRK-VII.2*	AT1G61390
G-LecRK-VIII	*AtG-LecRK-VIII.1*	AT1G61400
	*AtG-LecRK-VIII.2*	AT1G61420
	*AtG-LecRK-VIII.3*	AT1G61430
	*AtG-LecRK-VIII.4*	AT1G61440
	*AtG-LecRK-VIII.5*	AT1G61480
	*AtG-LecRK-VIII.6*	AT1G61490
	*AtG-LecRK-VIII.7*	AT1G61500
	*AtG-LecRK-VIII.8*	AT1G61550
Singletons	*AtG-LecRK-S.1*	AT1G11280
	*AtG-LecRK-S.2*	AT1G61360
	*AtG-LecRK-S.3*	AT1G61370
	*AtG-LecRK-S.4*	AT4G03230
	*AtG-LecRK-S.5*	AT4G11900

### Chromosomal location and prediction of Arabidopsis G-LecRK subcellular localization

The 38 Arabidopsis G-LecRKs were mapped onto the five chromosomes using the chromosomal map tool from TAIR. A single G-LecK locus, At1g11305, was not present on TAIR since it was created when At1g11300 was discovered as a misannotated gene and was split into At1g11300 and At1g11305 [70]. Therefore, the chromosomal location of At1g11305 was based on the location of At1g11300. Unlike Arabidopsis L-LecRKs, most of which are localized on chromosomes 5 and 3 [28, 30], the vast majority of the Arabidopsis G-LecRKs are localized on chromosome 1 (24 members), followed by chromosome 4 (eight members), chromosome 5 (three members), chromosome 2 (two members) and chromosome 3 (one member) (Additional file 7).

Arabidopsis G-LecRKs localization was predicted using SUBA3 [58, 59]. This tool predicted all Arabidopsis proteins to be localized at the plasma membrane, consistent with the existence of a TM domain. SUBA predictions were further investigated with TargetP 1.1 [60]. This tool predicts protein localization by analyzing cleavage site predictions and, therefore, predicts localization to the chloroplast, mitochondria or secretory pathways. Most of the Arabidopsis G-LecRKs were predicted to have a secretion signal peptide (Additional file 8). Two proteins, At1g61390 and At1g61400, were predicted to localize at the mitochondrial membrane and localization was not predicted by Target P 1.1 for one, At1g11280.

To validate localization predictions, the subCELlular LOcalization tool CELLO [61] was used. CELLO predictions mostly confirmed the predictions obtained by SUBA, but additionally revealed possible specific subcellular localization of two G-LecRKs, At4g27290 and At5g60900 (Additional file 8). These encode proteins without TMs, based on a domain search performed using Interpro, although both proteins were predicted to localize at the plasma membrane by SUBA. Interestingly, CELLO prediction added the possibility that these proteins could also localize to the nucleus and cytoplasm (Additional file 8).

### Kinase domain analysis of the tomato G-LecRKs

Like Arabidopsis, the presence of sites essential for catalytic activities of the 11 kinase subdomains [92] was investigated for the tomato G-LecRKs. The alignment of the tomato G-LecRKs kinase domains revealed overall conservation of the ATP-binding and catalytic sites, with a few substitutions in the other kinase subdomains (Additional file 9). The search also revealed ten genes with incomplete kinase domains with various amino acid modifications and indels in the subdomains (Table 4, Additional file 9). Additionally, Solyc07g063810 shows conservation of subdomains VI to XI, which includes the catalytic site, but displays several amino acid modifications in subdomains I to V, including the ATP binding site, suggesting it is likely an inactive kinase. Solyc03g063650 has a substitution of the aspartic acid to asparagine the kinase catalytic site, in subdomain VI, and lacks essential amino acids of subdomains I to IV suggesting it is also likely an inactive kinase (Additional file 9).

**Table 4:** Tomato G-LecRKs with incomplete kinase subdomains that lack some of the 11 subdomains

Tomato G-LecRK	Present kinase subdomains
Solyc04g008400.B	I and II
Solyc03g006780	I - V
Solyc04g008370	I - V
Solyc04g077300	I - V
Solyc08076070	I - V, VI
Solyc07g055630	I, II, VI - X
Solyc04g077380	I - V, VI, XI
Solyc07g055640.A	I - X
Solyc02g079710	I - X
Solyc07g063750	I - X

### Nomenclature for the tomato G-LecRKs

**Fig. 4:**
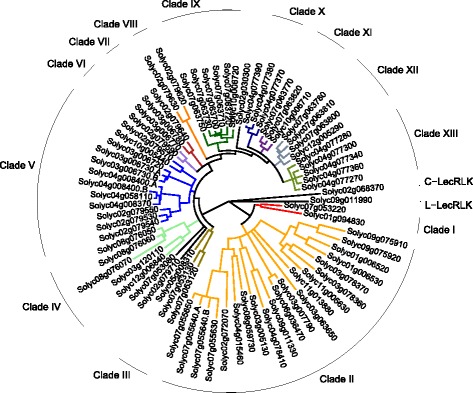
Maximum likelihood tree of amino acid sequences of G-LecRK sequences from tomato, with L-LecRK and C-LecRK sequences as outgroups. Labeled lines on the outside of the tree represent clade names as defined in the text, and clades are colored to match. Protein name suffixes (.A or .B) indicate that protein has been split apart for analysis as described in text. Nodes with < 70% bootstrap support were collapsed into polytomies. (Bootstrap percentages not shown)

Following a similar nomenclature as for the Arabidopsis G-LecRKs, the clades containing the 73 tomato G-LecRK members were used as the basis for naming the genes. This methodology resulted in the grouping of tomato G-LecRKs into 13 clades, within which genes are numbered progressively across chromosomes and clades (Fig. 4, Table 5, Additional file 10). Clades range in size from the largest, clade III, with 21 members, to four clades with two members each. Three genes do not fall in any of these clades and are designated singletons, two of which are on chromosome 2 and one on chromosome 7. As with Arabidopsis, chromosomal location is not correlated with clade membership.

**Table 5:** Classification and proposed nomenclature of the tomato G-LecRKs

Clade name	Gene name	Locus
G-LecRK-I	*SlG-LecRK-I.1*	Solyc01g094830
	*SlG-LecRK-I.2*	Solyc07g053220
G-LecRK-II	*SlG-LecRK-II.1*	Solyc01g006520
	*SlG-LecRK-II.2*	Solyc01g006530
	*SlG-LecRK-II.3*	Solyc02g072070
	*SlG-LecRK-II.4*	Solyc03g005130
	*SlG-LecRK-II.5*	Solyc03g007790
	*SlG-LecRK-II.6*	Solyc03g063650
	*SlG-LecRK-II.7*	Solyc03g078370
	*SlG-LecRK-II.8*	Solyc03g078360
	*SlG-LecRK-II.9*	Solyc04g015460
	*SlG-LecRK-II.10*	Solyc04g078410
	*SlG-LecRK-II.11*	Solyc06g036470
	*SlG-LecRK-II.12*	Solyc07g055630
	*SlG-LecRK-II.13*	Solyc07g055640.A
	*SlG-LecRK-II.14*	Solyc07g055640.B
	*SlG-LecRK-II.15*	Solyc07g055650
	*SlG-LecRK-II.16*	Solyc08g059730
	*SlG-LecRK-II.17*	Solyc09g011330
	*SlG-LecRK-II.18*	Solyc09g075910
	*SlG-LecRK-II.19*	Solyc09g075920
	*SlG-LecRK-II.20*	Solyc11g005630
	*SlG-LecRK-II.21*	Solyc11g013880
G-LecRK-III	*SlG-LecRK-III.1*	Solyc05g008310
	*SlG-LecRK-III.2*	Solyc07g053120
	*SlG-LecRK-III.3*	Solyc07g053130
G-LecRK-IV	*SlG-LecRK-IV.1*	Solyc03g120110
	*SlG-LecRK-IV.2*	Solyc08g076050
	*SlG-LecRK-IV.3*	Solyc08g076060
	*SlG-LecRK-IV.4*	Solyc08g076070
	*SlG-LecRK-IV.5*	Solyc12g006840
G-LecRK-V	*SlG-LecRK-V.1*	Solyc02g079530
	*SlG-LecRK-V.2*	Solyc02g079540
	*SlG-LecRK-V.3*	Solyc02g079550
	*SlG-LecRK-V.4*	Solyc02g079570
	*SlG-LecRK-V.5*	Solyc03g006720
	*SlG-LecRK-V.6*	Solyc03g006730.A
	*SlG-LecRK-V.7*	Solyc03g006730.B
	*SlG-LecRK-V.8*	Solyc04g008370
	*SlG-LecRK-V.9*	Solyc04g008400.A
	*SlG-LecRK-V.10*	Solyc04g008400.B
	*SlG-LecRK-V.11*	Solyc04g058110
	*SlG-LecRK-V.12*	Solyc10g005440
G-LecRK-VI	*SlG-LecRK-VI.1*	Solyc02g079590
	*SlG-LecRK-VI.2*	Solyc03g006770
G-LecRK-VII	*SlG-LecRK-VII.1*	Solyc02g079640
	*SlG-LecRK-VII.2*	Solyc03g006780
G-LecRK-VIII	*SlG-LecRK-VIII.1*	Solyc02g079620
	*SlG-LecRK-VIII.2*	Solyc02g079630
G-LecRK-IX	*SlG-LecRK-IX.1*	Solyc07g063700
	*SlG-LecRK-IX.2*	Solyc07g063710
	*SlG-LecRK-IX.3*	Solyc07g063720
	*SlG-LecRK-IX.4*	Solyc07g063730
	*SlG-LecRK-IX.5*	Solyc07g063750
	*SlG-LecRK-IX.6*	Solyc10g006720
G-LecRK-X	*SlG-LecRK-X.1*	Solyc04g077370
	*SlG-LecRK-X.2*	Solyc04g077380
	*SlG-LecRK-X.3*	Solyc04g077390
G-LecRK-XI	*SlG-LecRK-XI.1*	Solyc07g063770
	*SlG-LecRK-XI.2*	Solyc07g063820
	*SlG-LecRK-XI.3*	Solyc10g006710
G-LecRK-XII	*SlG-LecRK-XII.1*	Solyc07g063780
	*SlG-LecRK-XII.2*	Solyc07g063800
	*SlG-LecRK-XII.3*	Solyc07g063810
	*SlG-LecRK-XII.4*	Solyc12g005290
G-LecRK-XIII	*SlG-LecRK-XIII.1*	Solyc04g077270
	*SlG-LecRK-XIII.2*	Solyc04g077280
	*SlG-LecRK-XIII.3*	Solyc04g077300
	*SlG-LecRK-XIII.4*	Solyc04g077340
	*SlG-LecRK-XIII.5*	Solyc04g077360
Singletons	*SlG-LecRK-S.1*	Solyc02g030300
	*SlG-LecRK-S.2*	Solyc02g079710
	*SlG-LecRK-S.3*	Solyc07g053080

### Chromosomal location and prediction of tomato G-LecRKs subcellular localization

The 73 G-LecRK loci were mapped onto the 12 tomato chromosomes. As for Arabidopsis, locations of the genes that were misannoted and split into 2, were based on the location of the original locus. Members of tomato G-LecRKs are distributed throughout the 12 tomato chromosomes, with over half (54%) localized on chromosome 7, 4, 2, and 3 encompassing 18, 14, 11, and 11 members, respectively (Additional file 11) [30]. In contrast, tomato L-LecRKs are mostly localized on chromosome 9 and 10, with members located on 8 of the 12 chromosomes.

Tomato G-LecRK protein sequences were used to predict subcellular localization with TargetP 1.1 [60]. The localization of tomato C-LecRKs and L-LecRKs have not been investigated. However L-LecRKs possess TM domains and are predicted to localize mostly at the plasma membrane with a few members predicted to localize to mitochondria or chloroplast [28]. TargetP predicted that the great majority of tomato G-LecRKs have secretion pathway signals and are localized to the plasma membrane (Additional file 12). A single protein (Solyc02g079630) was predicted to have chloroplast localization. Four proteins (Solyc03g006730.B, Solyc07g055640.A, Solyc07g063810 and Solyc11g005630) were predicted to localize to mitochondria. Eight proteins (Solyc07g055640.B, Soly04g077380, Solyc08g076060, Solyc07g055650, Solyc07g055630, Solyc04g008400.B, Solyc02g030300 and Solyc08g076070), for which a signal peptide could not be predicted using this tool, were not localized to a specific subcellular compartment. Additionally, the subcellular localization tool, CELLO, was used to investigate the localization of the tomato G-LecRKs and showed an overlap of prediction of plasma membrane localization and presence of TargetP secretion pathway signal. Interestingly, this tool was able to predict subcellular localization of proteins for which TargetP could not predict localization, and was also able to predict membrane localization for proteins that did not have a predicted TM domain, suggesting a different membrane-associated signal for these proteins. CELLO predictions also suggested multiple localizations for a few tomato G-LecRKs (such as Solyc01g006530 and Solyc07g055630) and contradicted a few predictions by TargetP (such as Solyc02g079630 and Solyc03g006730.B; Additional file 12).

### Comparison of conserved motifs in ectodomains of tomato and Arabidopsis G-LecRKs

**Fig. 5:**
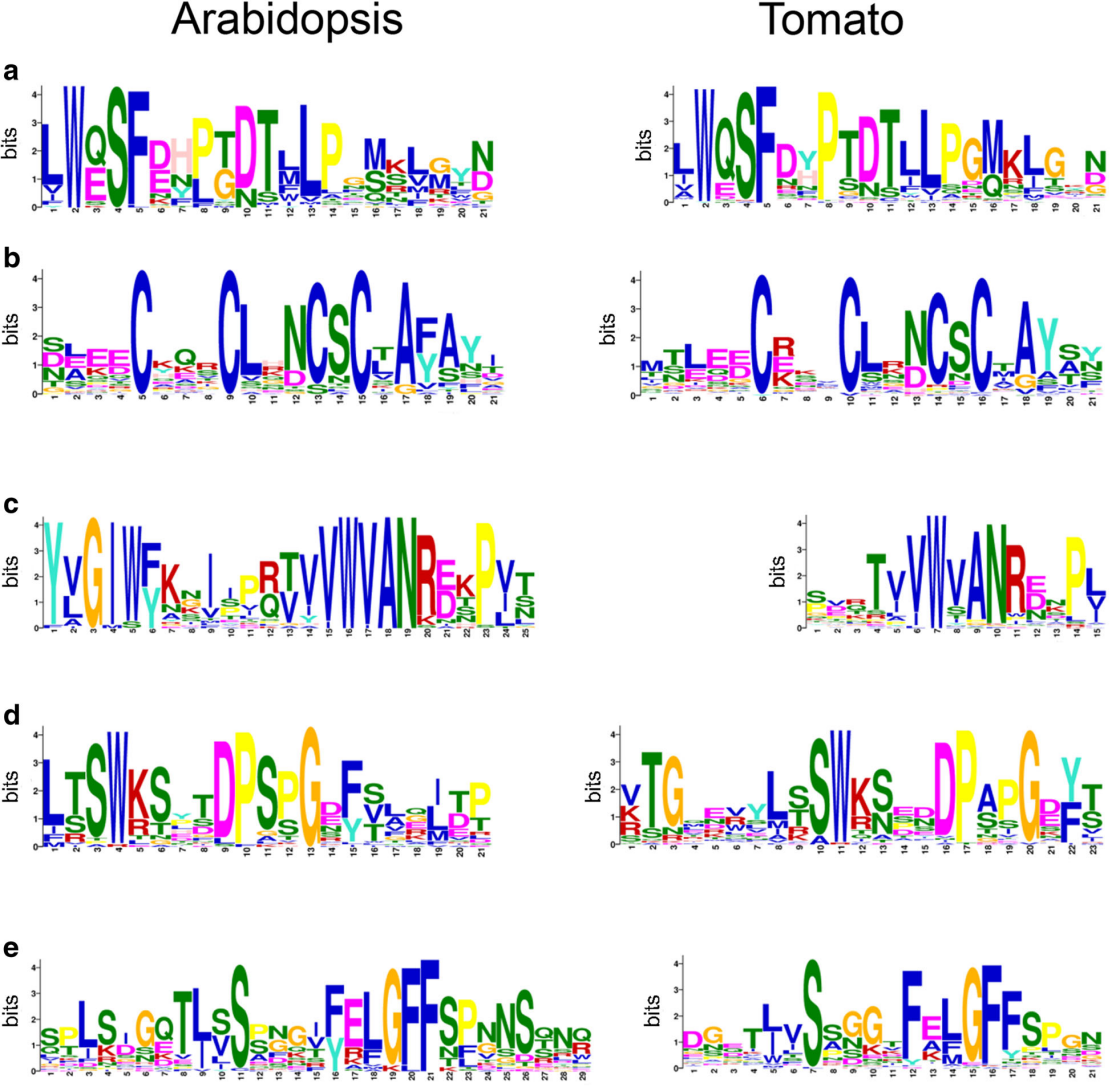
Conserved motifs in extracellular domains of Arabidopsis and tomato G-LecRKs. Motifs presented (**a**-**e**) were identified using MEME. Each column in the x-axis is composed of stack of letters where the height of these letters is indicative of the frequency of the letter at that position. The height of the stack is indicative of the sequence conservation

The predicted cytoplasmic-localized regions of G-LecRKs consist of the extremely conserved kinase domains. To investigate the presence of conserved motifs in the ectodomain of the Arabidopsis and tomato G-LecRKs, the amino acid sequences of the ectodomains were submitted to MEME [57]. Despite the high variability of the ectodomains, five motifs present in at least 30 of the 38 Arabidopsis sequences and in at least 45 of the 73 tomato sequences were identified (Fig. 5).

The highest conserved ectodomain motif (Fig. 5a) was present in all Arabidopsis and tomato G-LecRKs and it was previously shown to be present in 96% of the rice G-LecRKs [28]. One of the motifs, a cysteine-rich region within the PAN domain (Fig. 5b), is present in 34 and 66 of the Arabidopsis and tomato G-LecRKs, respectively. Interestingly, a third motif (Fig. 5c) is also conserved in 76 out of 100 rice G-LecRKs, and was previously identified in 27 Arabidopsis G-LecRKs [28]. The two remaining ectodomain motifs are novel and have not been reported previously (Fig. 5d, e). The conservation of the motifs in the ectodomain of both Arabidopsis and tomato G-LecRKs is remarkable considering that these extracellular domains harbor the lectin domain known to have low conservation among members of this family from a single plant species [28].

## Discussion

Two previous studies have reported Arabidopsis to have 32 [28, 93] G-LecRK members, a number lower than the 38 members identified in this study. One of the possible reasons for this discrepancy from Shiu and Bleecker [93], is the current improved annotation of the Arabidopsis genome. Their study also used the presence of a TM domain as a criterion for their analysis, but this was not used in our study. As for the lower number identified by Vaid et al. [28], this could be due to the fact that their analysis relied on sequence similarity to a single gene sequence, At1g61610, while in our analysis, we used a number of candidate sequences from our initial search results to fish out additional candidates. Nevertheless, their overall criterion for candidates was the same as the one used here, which is the presence of both lectin and kinase domains.

Our search retrieved all genes identified by Vaid et al. [28] and an additional 16 sequences that have a G-type lectin domain. Their gene list included a single gene, At1g61460, that is not present on our list and which does not encode a G-type lectin domain, so was not recovered in our BLASTp searches. Of the 16 new sequences with a G-type lectin domain, nine do not encode a kinase domain and would not have been retrieved by Vaid et al. [28]. These were also excluded from our analysis. Taken together, our results added seven proteins to the previous list of Arabidopsis G-LecRKs. Of these seven additional genes, At1g67520 and At5g24080 encode proteins with atypical kinase domains and lacking several subdomains, while At2g41890 lacked essential amino acids at the ATP binding site and the catalytic loop, suggesting they are defective kinases [34]. While kinase activity could be crucial for the function of these proteins, lacking kinase activity may not abolish function, as function for kinase inactive receptor-like kinases has been previously reported [94, 95].

The same search methodology used for identifying members of this family in Arabidopsis showed success with identification of the members in tomato. These investigations of the tomato genome allowed the identification of 73 genes encoding proteins with both a kinase domain and a G-lectin domain and revealed expansion of the number of members of this family in tomato relative to Arabidopsis. As in Arabidopsis, several of the 73 tomato G-LecRKs have mutations and deletions in their kinase domains and seem to be inactive kinases, indicative of functional diversification.

Phylogenetic analysis of G-LecRK sequences consistently shows the genes falling into two clades, whether one, three, or four species is included. One of these clades includes the L-LecRK sequences intended as outgroup along with the C-LecRKs. This configuration would indicate that the L-type genes should be merged into the G-type, because sequence analysis shows they are not distinct. However, Arabidopsis alone has 45 L-LecRKs and this analysis included only one, therefore additional analyses are needed to elucidate the relationship between G- and L-LecRKs. The division of the G-type genes into two clades, each with high support, also suggests that this group could be subdivided into two different LecRK groups, however, based on current evidence, there does not seem to be functional differentiation between the two clades, therefore it is reasonable to consider them a single group for purposes of understanding LecRK function and evolution.

There is no pattern discernable in the expression and function data currently available regarding G-LecRKs. Whereas all recorded functions and expression data are consistent with roles in biotic and abiotic stress responses or developmental processes, which involve similar pathways, no clade seems specialized for specific functions within these categories. It is not possible to say “there is a clade of genes that responds to trehalose, and it has expanded in species X, therefore trehalose response is important in species X.” Rather, we find closely related genes that appear to have taken on different functions related to stress response and development. Clade A2b2, which includes the largest cluster of Arabidopsis genes, includes members with putative functions ranging from gravitropism to pollen and root hair development to resistance to bacterial infection [71, 75, 76, 84, 85, 96]. Clades with fewer Arabidopsis sequences, such as B2b1 with two, still show variation, in this case one gene that responds to gravity and one that responds to water loss [72, 74]. Two points are important to keep in mind when evaluating these expression and function data, however. First, data are only available for genes from Arabidopsis; we currently do not have data for tomato or columbine genes. Second, the current analyses are based on published reports, but it is highly likely that no study tested all possible biotic and abiotic factors. Therefore the fact that At1g11340.1 is suppressed by mevalonic acid does not mean it might not be up- or downregulated in response to other factors [73].

Analysis of the clades in the phylogenetic tree shows that there has been species-specific gene expansion in different clades across the tree, and examination of chromosomal locations of the genes suggests combinations of tandem and possible whole genome duplications. Notably, clade B has only 7 Arabidopsis genes, and no subclade has more than two. In contrast, clade B has 23 tomato genes, with one subclade that has a single Arabidopsis gene (B2b2) having 7 tomato genes. This same subclade has 5 columbine genes. Clade B1 has one gene each from Arabidopsis and tomato, but 9 from columbine. Clade A has a greater number of genes from Arabidopsis, but again we see species-specific expansion in different subclades in all three species. Clade A2b2 has 3 tomato genes, which form a sister-group to 13 Arabidopsis genes; this is the largest Arabidopsis gene group in the tree. The tomato genes are all located on chromosome 3, and the Arabidopsis genes are all located on chromosome 1, suggesting a single ancestral gene that underwent tandem duplications in each species independently, and to a greater extent in Arabidopsis. Clade A1 consists of three subclades, two of which have genes only from Arabidopsis and tomato, and one of which has genes from all three. This topology implies three ancestral genes, with loss of columbine genes from the first two clades. These clades (A1a, A1b) further show evidence of additional duplications within tomato and Arabidopsis, although in this case these appear potentially to be a combination of tandem and whole genome duplications based on chromosomal locations. The third clade, A1c, again shows dramatic expansion in columbine and especially tomato, but not Arabidopsis. Because there is no clear pattern of differential function or expression among clades, it is possible that the differential diversification of clades in different species is essentially stochastic. Duplication appears to be extremely common, and the extent in each clade in each species may not be specifically under selection. In fact, the independent expansion and relatively small number of clades with genes from both tomato and Arabidopsis suggests that the base number of core eudicot G-LecRKs is low, and that the differences in numbers between the two species is more a result of expansion than loss. This is also consistent with the role of these genes in stress responses.

While clustering of G-LecRKs members on chromosomes, such as Arabidopsis chromosome 1 and tomato chromosome 7, suggests duplications, our analysis indicates that chromosomal location is not predictive of a clade membership for either plant species. This suggests that G-LecRKs are rapidly evolving and diversifying consistent with their known functional roles in biotic and abiotic stress responses and development.

Clade membership was also not indicative of the presence of specific ectodomain configurations, specifically presence (or absence) of three domains: SLG, EGF and PAN. The importance of each of these domains, as well as their contributions to G-LecRK activity, have not been investigated to date. Nevertheless, it is to be expected that relevant regions at the ectodomain, outside of the region that confers substrate-binding specificity, would be conserved among different members of the same family. Consistent with this hypothesis, a motif search among members from Arabidopsis and tomato revealed the presence of a single motif (Fig. 5a) in all members of G-LecRKs from both plant species. This motif was also identified in a previous investigation in both Arabidopsis and rice (96% of rice G-LecRKs) [28]. Interestingly, the second motif identified in our search (Fig. 5b), present in 34 Arabidopsis G-LecRKs and 66 tomato G-LecRKs was also identified in 76% of the rice G-LecRKs by the same authors. The observation that the lectin domain is the domain with low conservation in G-LecRKs and the presence of conserved motifs in the ectodomain shows that despite the lack of conservation of the lectin domain, a specific motif is conserved and might constitute essential site(s) for protein activity. The two newly identified motifs, observed in 30 of the 38 Arabidopsis and 45 of the 73 tomato lectin domain of G-LecRKs, are less common. Their presence in additional plant species and how widespread these new motifs are remains to be investigated.

## Conclusions

We present here the results of an analysis of G-LecRK gene lineage evolution in Arabidopsis, a member of the mustard family (Brassicaceae, in the rosid clade) and tomato, a member of the nightshade family (Solanaceae, in the asterid clade). Given that these are both members of the derived angiosperm clade, core eudicots, we also included an evaluation of G-LecRKs in columbine (*Aquilegia coerulea*), a member of the basal eudicots that diverged before the rosid-asterid split in the core eudicots to polarize the tree and allow analysis of duplication, expansion, and loss of G-LecRK genes and gene clades.

The present investigation added to the number of currently known Arabidopsis G-LecRKs and characterized for the first time the tomato G-LecRKs. We proposed a nomenclature for both Arabidopsis and tomato G-LecRKs and identified possible essential sites for G-LecRK activity. Additionally, prediction of protein localization by different tools enriched the initial prediction of G-LecRKs plasma membrane localization and raised the possibility for specificity of modes of actions of a number of G-LecRKs depending on their specific subcellular localization patterns. Given their putative roles in plant defense, and the importance of tomato as a crop, an understanding of the structure and evolution of these proteins in tomato may shed light on defense strategies that can be leveraged to produce hardier plants and yield.

## Additional files

Additional file 1: Members of the columbine G-lectins and their domains. (XLSX 57 kb)

Additional file 2: Members of the rice G-lectins and their domains. (XLS 33 kb)

Additional file 3: Maximum likelihood tree of amino acid sequences from G-LecRK, L-LecRK intended outgroups, and C-LecRK outgroups from tomato, Arabidopsis, columbine, and rice. Bootstrap support from 1000 replicates is shown above nodes. Brackets on the right indicate intended outgroup clades. (PDF 20 kb)

Additional file 4 Maximum likelihood tree of amino acid sequences from G-LecRK, L-LecRK intended outgroups, and C-LecRK outgroups from tomato, Arabidopsis, and columbine. Bootstrap support from 1000 replicates is shown above nodes. Brackets on the right indicate the major clades as defined in the text along with the two intended outgroup clades. (PDF 19 kb)

Additional file 5: Alignment of predicted amino acid sequences of Arabidopsis G-LecRKs kinase domains with the L-LecRK-VI.2 using ClustalW. Lines on top of the alignment show subdomains I and II, ATP binding site (GxGxxGxV) and subdomain VI, the serine/threonine kinase active site (HRDLKxxN). (PDF 3979 kb)

Additional file 6: Maximum likelihood tree of amino acid sequences from G-LecRK, L-LecRK outgroups, and C-LecRK outgroups from Arabidopsis. Bootstrap support from 1000 replicates is shown above nodes. (PDF 11 kb)

Additional file 7: Genetic map of the Arabidopsis G-LecRKs. Arrangement of G-LecRKs on the five Arabidopsis chromosomes. Figure was prepared using Chromosome Map Tool in TAIR. Locus At1g11305 was added manually. (TIFF 337 kb)

Additional file 8: Subcellular localization of the Arabidopsis G-LecRKs. Localization was predicted using SUBA, TargetP and CELLO software programs/tools. (PDF 55 kb)

Additional file 9: Alignment of predicted amino acid sequences of tomato G-LecRKs kinase domains with Solyc03g006720 using ClustalW. Lines on top of alignment show subdomains I and II, ATP binding site (GxGxxGxV) and subdomain VI, the serine/threonine kinase active site (HRDLKxxN). (PDF 7110 kb)

Additional file 10: Maximum likelihood tree of amino acid sequences from G-LecRK, L-LecRK outgroups, and C-LecRK outgroups from tomato. Bootstrap support from 1000 replicates is shown above nodes. (PDF 7 kb)

Additional file 11: Genetic map of the tomato G-LecRKs. Arrangement of G-LecRKs on the 12 tomato chromosomes. Figure was prepared manually using map viewer in NCBI. (TIFF 309 kb)

Additional file 12: Subcellular localization of the tomato G-LecRKs. Localization was predicted using TargetP and CELLO software programs/tools. (PDF 56 kb)

## Abbreviations

C-type: Calcium-dependent
EGF: Epidermal growth factor
G-type: *Galanthus nivalis* agglutinin type
LecRKs: Lectin receptor kinases
LPS: Lipopolysaccharide
LRR: Leucine-rich repeat
L-type: Legume-like
PAN: Plasminogen-apple-nematode
PRR: Pattern-recognition receptors
*Pst*: *Pseudomonas syringae* pv. *tomato*
RK: Receptor kinase
SLG: S-locus glycoprotein
TM: Transmembrane
