# Supplementary figures and images for: Classification and phylogenetic analyses of the Arabidopsis and tomato G-type lectin receptor kinases
Source: BMC Genomics. 2018 Apr 6;19:239. doi: 10.1186/s12864-018-4606-0 (PMC5889549; doi:10.1186/s12864-018-4606-0)

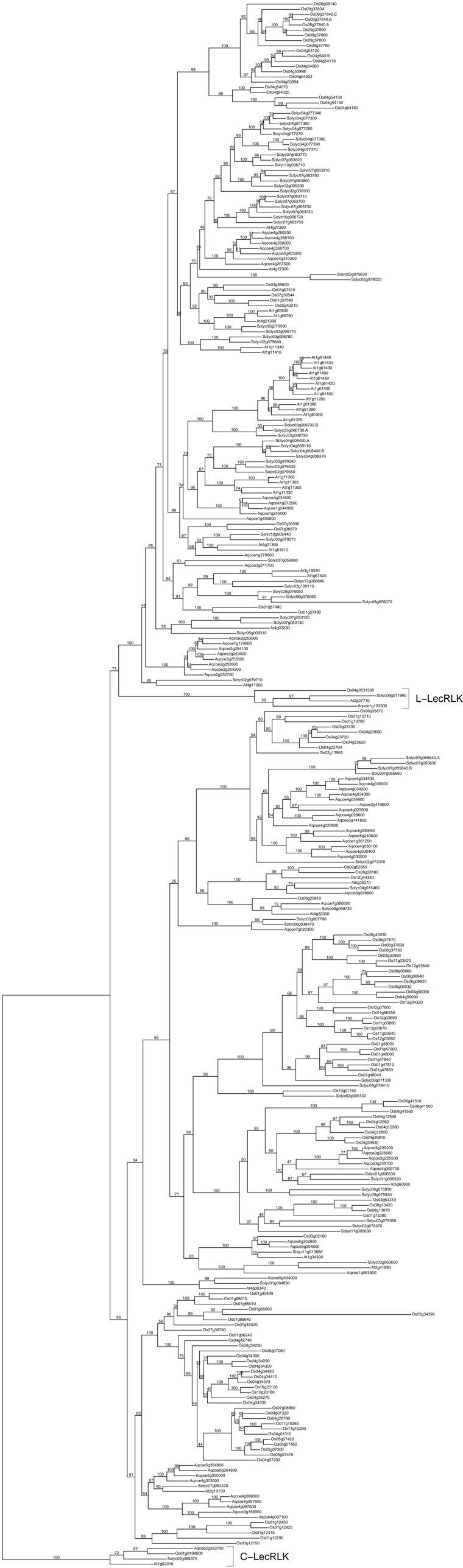

L-LecRLK

C-LecRLK

Supplement: Supplementary file 3 — Maximum likelihood tree of amino acid sequences from G-LecRK, L-LecRK intended outgroups, and C-LecRK outgroups from tomato, Arabidopsis, columbine, and rice. Bootstrap support from 1000 replicates is shown above nodes. Brackets on the right indicate intended outgroup clades. (PDF 20 kb) [file 12864_2018_4606_MOESM3_ESM.pdf]

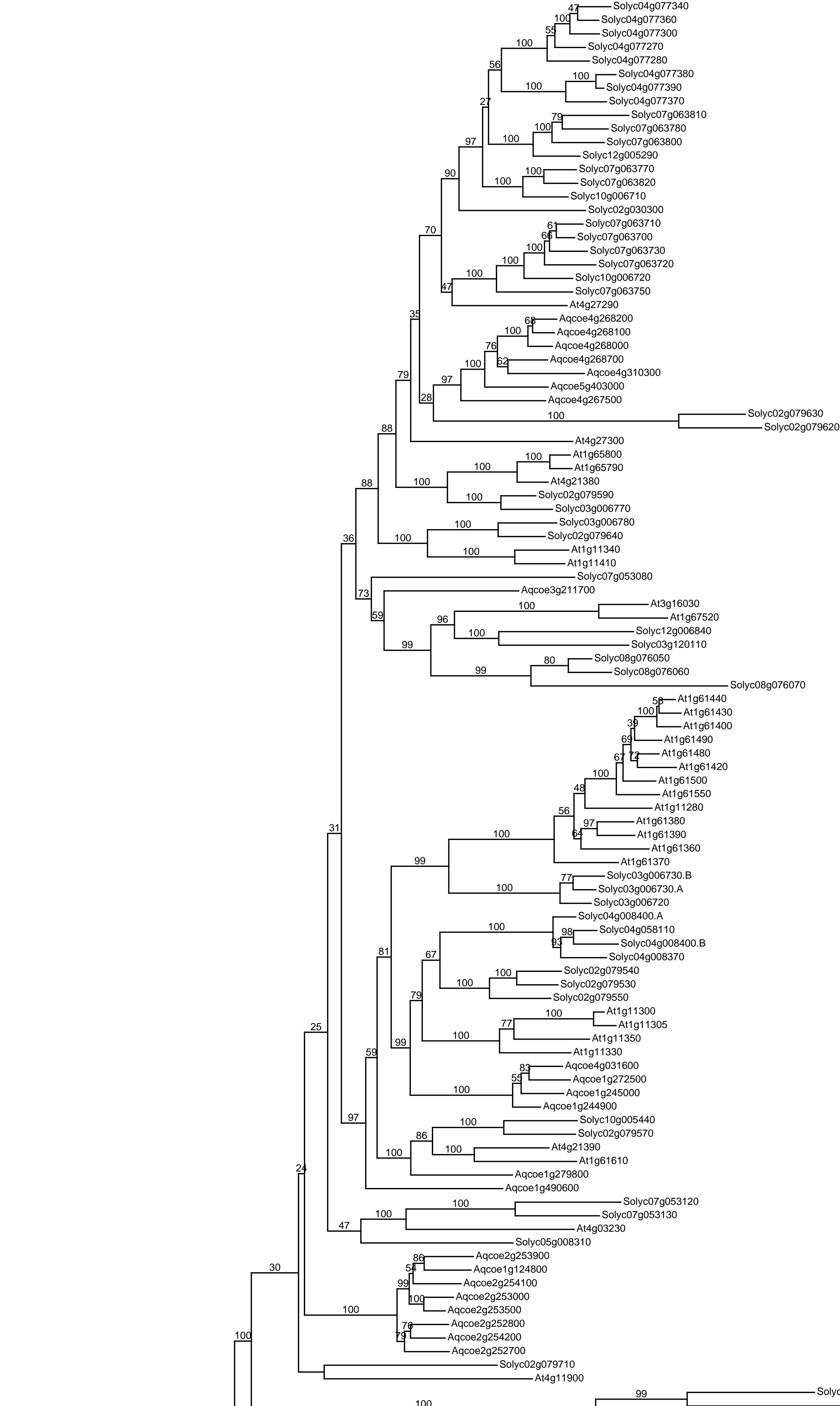

A

L-LecRLK

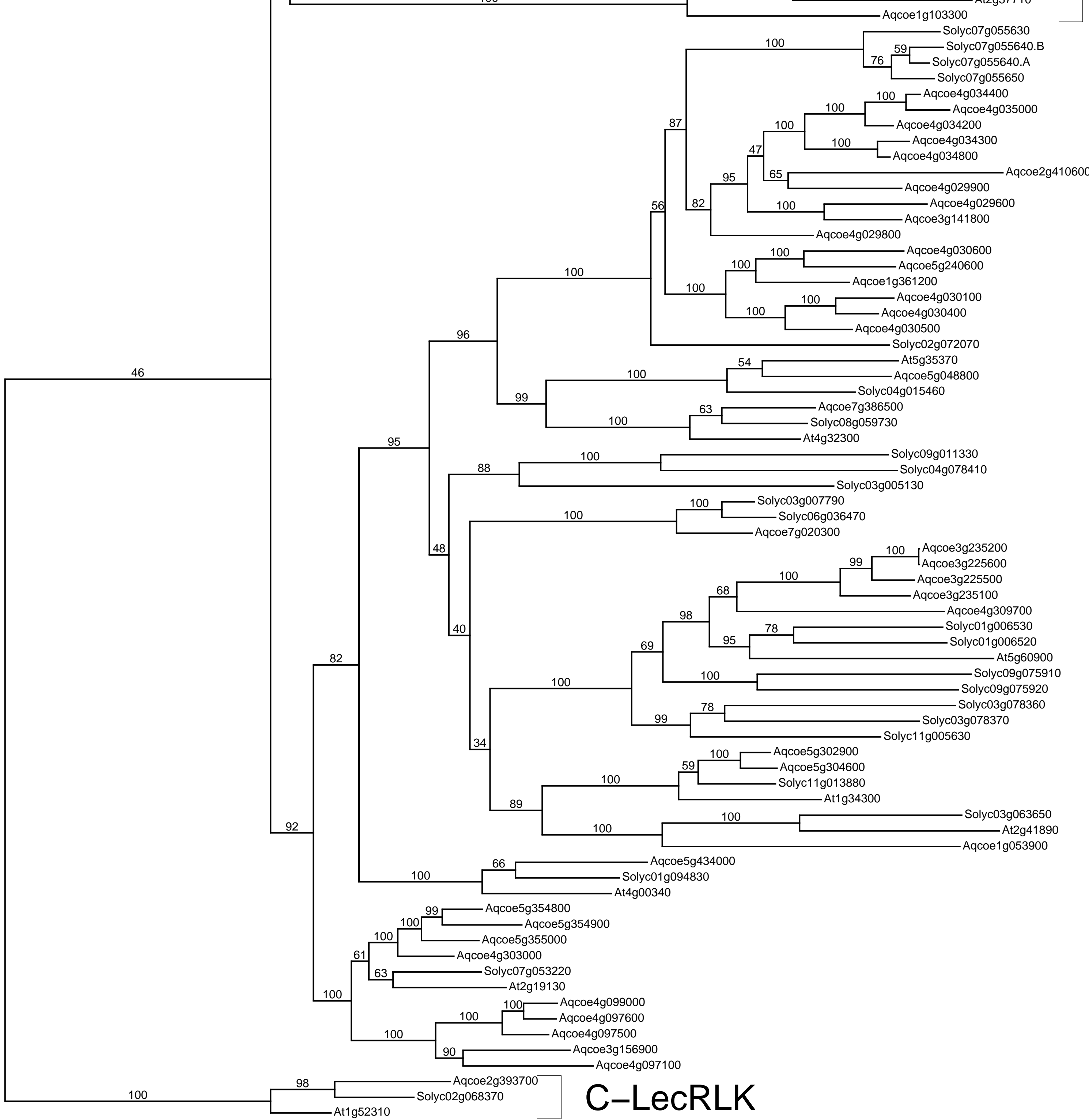

B

C-LecRLK

Supplement: Supplementary file 4 — Maximum likelihood tree of amino acid sequences from G-LecRK, L-LecRK intended outgroups, and C-LecRK outgroups from tomato, Arabidopsis, and columbine. Bootstrap support from 1000 replicates is shown above nodes. Brackets on the right indicate the major clades as defined in the text along with the two intended outgroup clades. (PDF 19 kb) [file 12864_2018_4606_MOESM4_ESM.pdf]

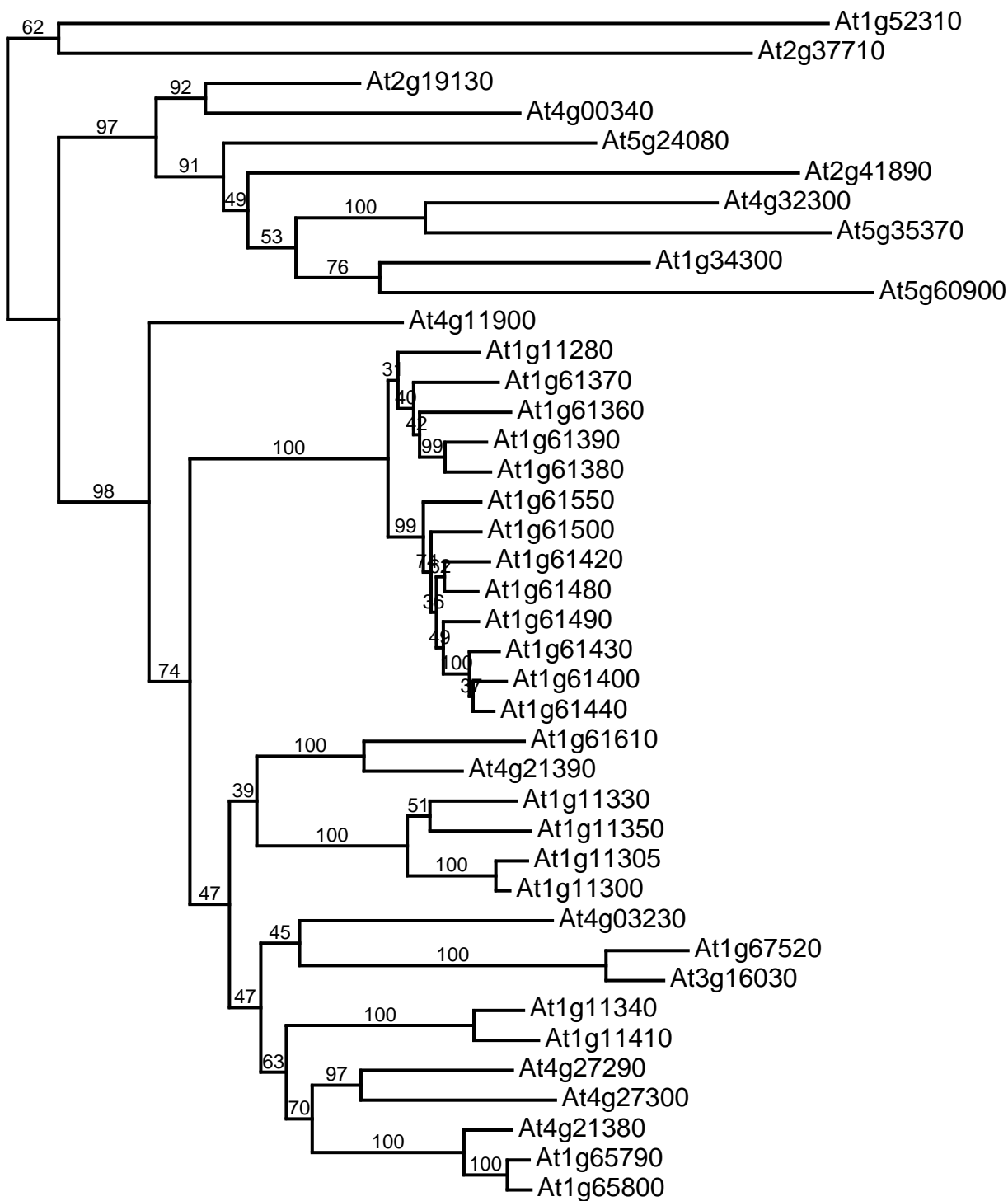

Supplement: Supplementary file 6 — Maximum likelihood tree of amino acid sequences from G-LecRK, L-LecRK outgroups, and C-LecRK outgroups from Arabidopsis. Bootstrap support from 1000 replicates is shown above nodes. (PDF 11 kb) [file 12864_2018_4606_MOESM6_ESM.pdf]

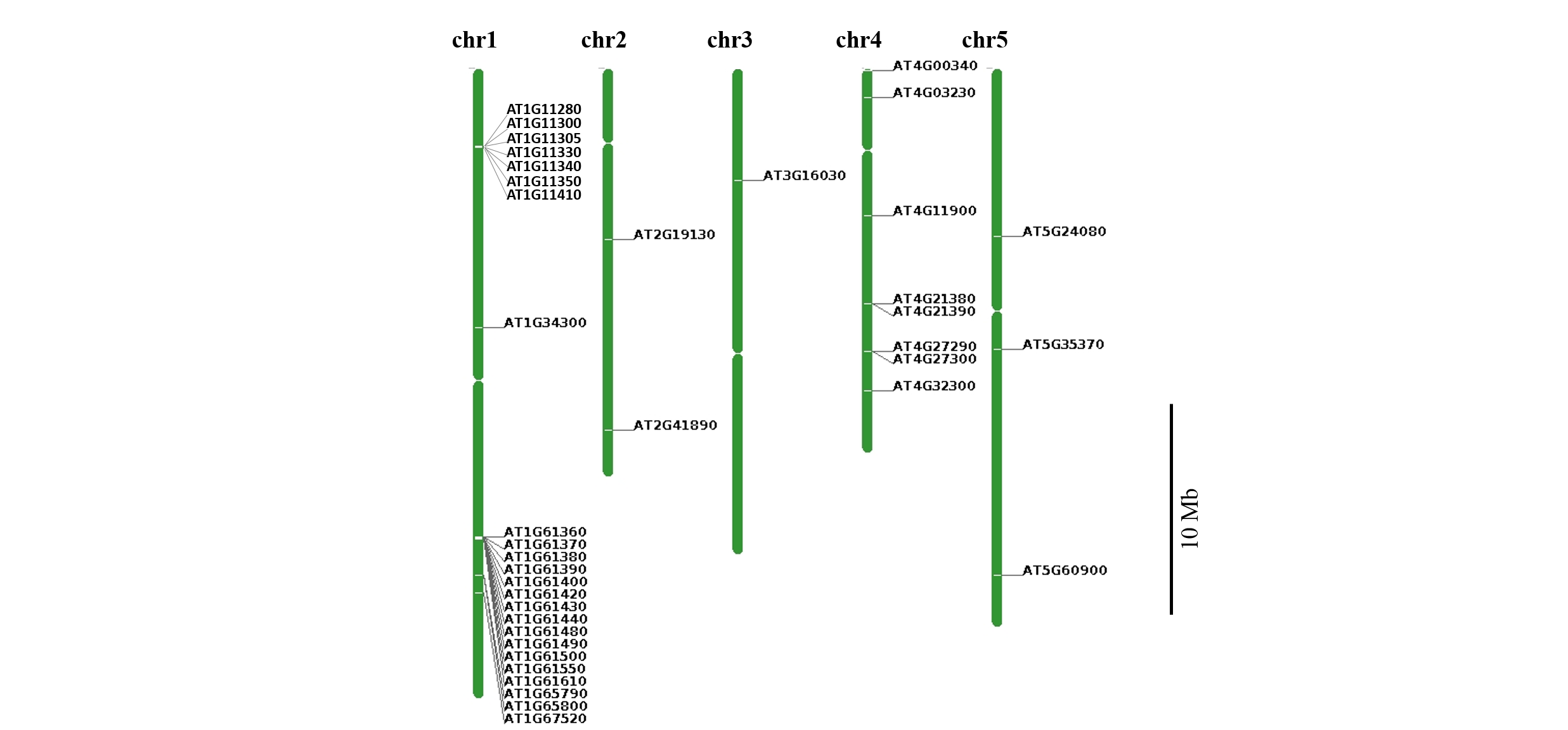

Supplement: Supplementary file 7 — Genetic map of the Arabidopsis G-LecRKs. Arrangement of G-LecRKs on the five Arabidopsis chromosomes. Figure was prepared using Chromosome Map Tool in TAIR. Locus At1g11305 was added manually. (TIFF 337 kb) [file 12864_2018_4606_MOESM7_ESM.tiff]

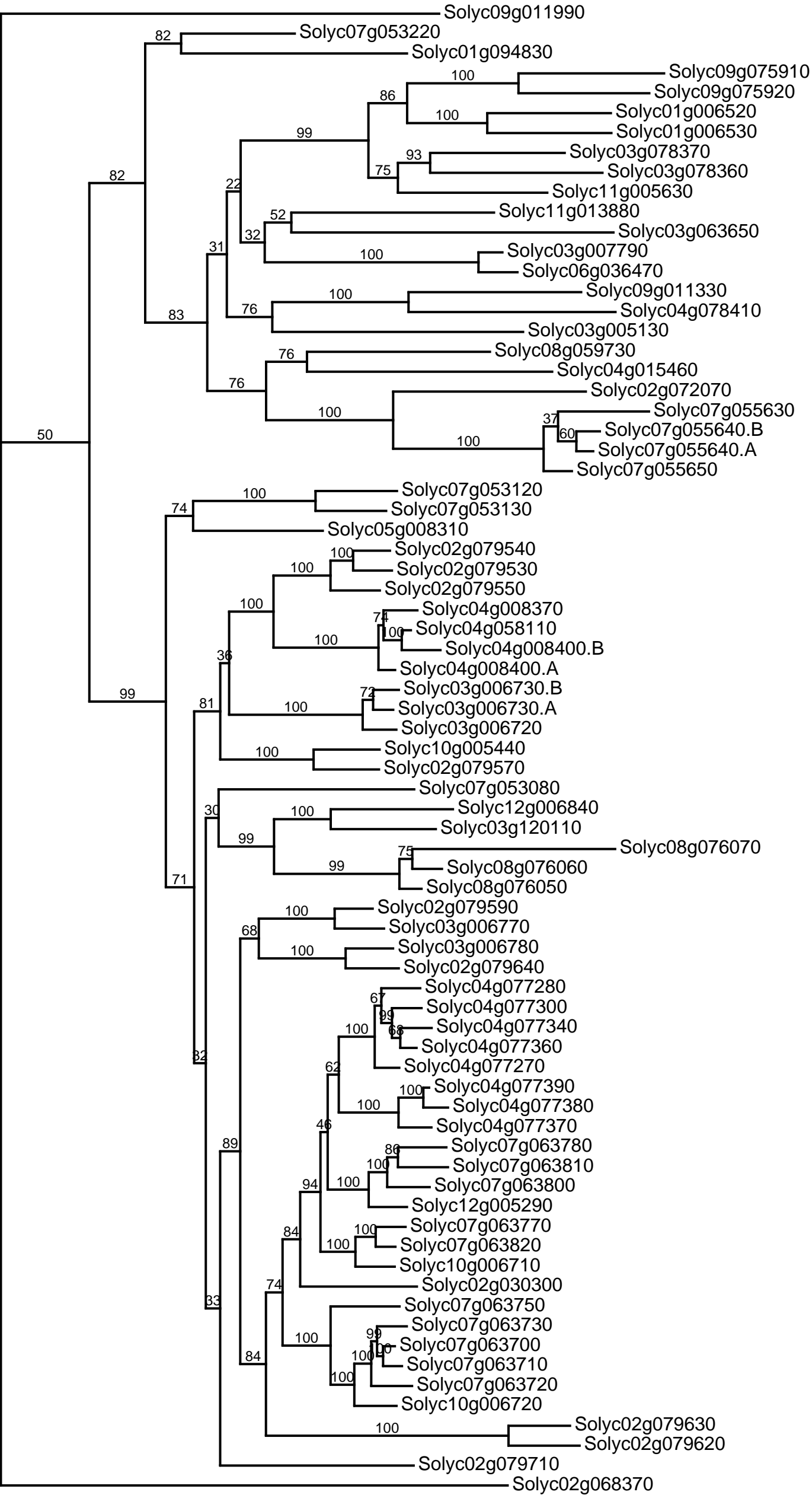

Supplement: Supplementary file 10 — Maximum likelihood tree of amino acid sequences from G-LecRK, L-LecRK outgroups, and C-LecRK outgroups from tomato. Bootstrap support from 1000 replicates is shown above nodes. (PDF 7 kb) [file 12864_2018_4606_MOESM10_ESM.pdf]

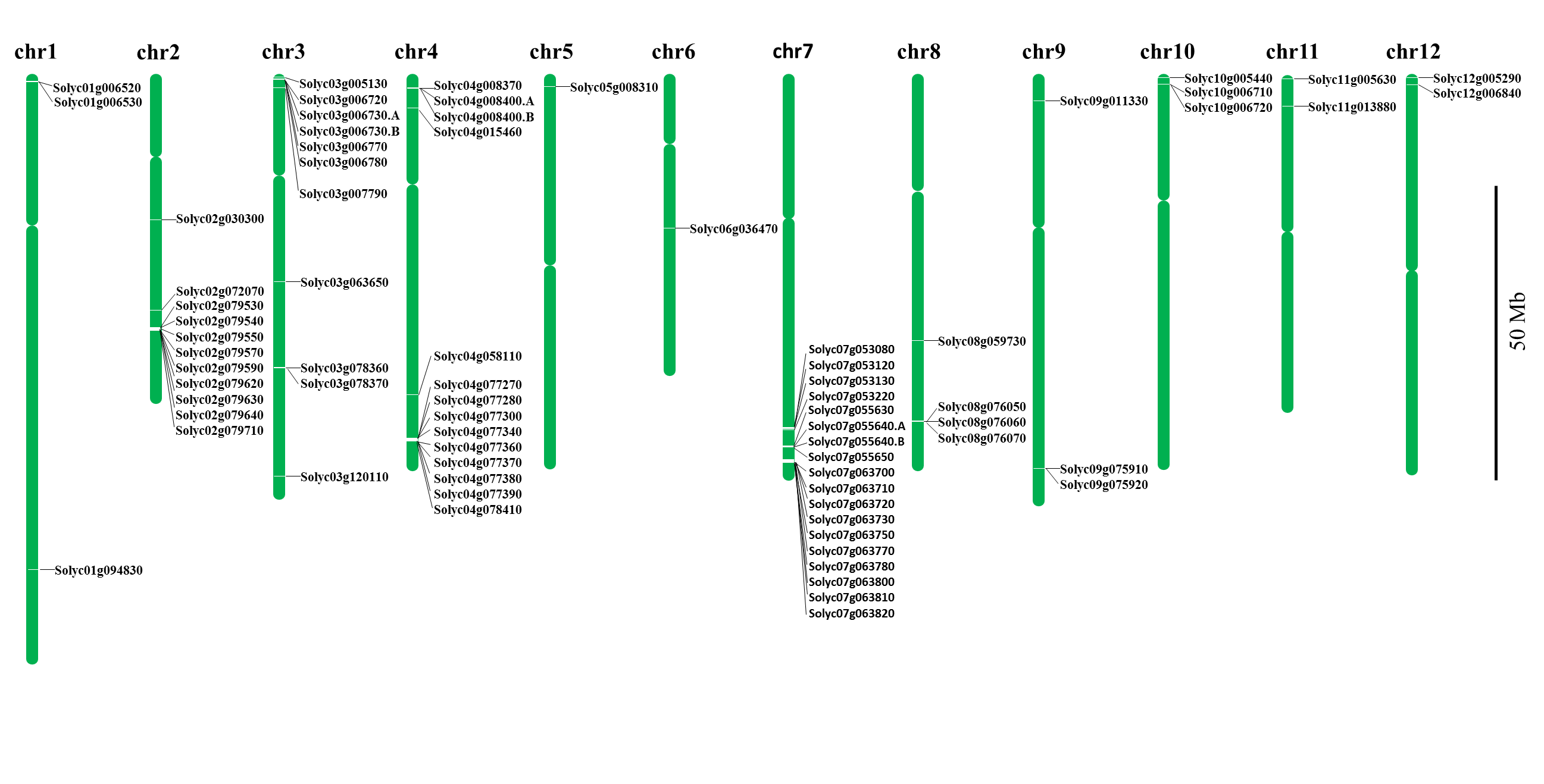

Supplement: Supplementary file 11 — Genetic map of the tomato G-LecRKs. Arrangement of G-LecRKs on the 12 tomato chromosomes. Figure was prepared manually using map viewer in NCBI. (TIFF 309 kb) [file 12864_2018_4606_MOESM11_ESM.tiff]
