# Supplementary material for: Classification and phylogenetic analyses of the Arabidopsis and tomato G-type lectin receptor kinases
Source: BMC Genomics. 2018 Apr 6;19:239. doi: 10.1186/s12864-018-4606-0 (PMC5889549; doi:10.1186/s12864-018-4606-0)
Supplement: Supplementary file 5 — Alignment of predicted amino acid sequences of Arabidopsis G-LecRKs kinase domains with the L-LecRK-VI.2 using ClustalW. Lines on top of the alignment show subdomains I and II, ATP binding site (GxGxxGxV) and subdomain VI, the serine/threonine kinase active site (HRDLKxxN). (PDF 3979 kb) [file 12864_2018_4606_MOESM5_ESM.pdf]

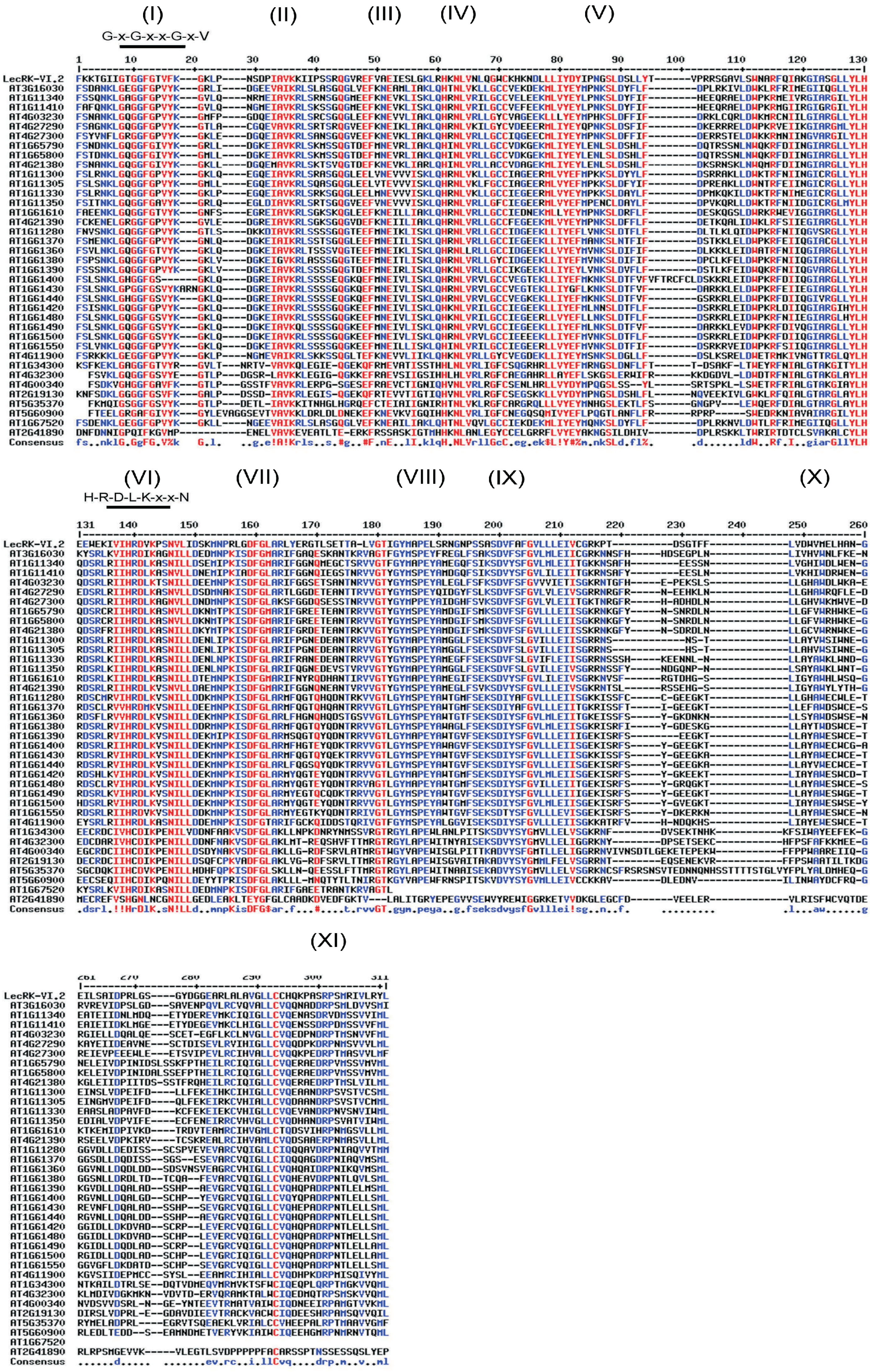
(I)
G-x-G-x-x-G-x-V
(II)
(III)
(IV)
(V)
(VI)
H-R-D-L-K-x-x-N
(VII)
(VIII)
(IX)
(X)
(XI)
