# Supplementary material for: Classification and phylogenetic analyses of the Arabidopsis and tomato G-type lectin receptor kinases
Source: BMC Genomics. 2018 Apr 6;19:239. doi: 10.1186/s12864-018-4606-0 (PMC5889549; doi:10.1186/s12864-018-4606-0)
Supplement: Supplementary file 8 — Subcellular localization of the Arabidopsis G-LecRKs. Localization was predicted using SUBA, TargetP and CELLO software programs/tools. (PDF 55 kb) [file 12864_2018_4606_MOESM8_ESM.pdf]

Additional file 8. Subcellular localization of the Arabidopsis G-LecRKs. Localization was predicted using SUBA, TargetP and CELLO software programs/tools.

	Locus	SUBA	TargetP	CELLO
**1**	AT1G11280	PM^	-*	PM
**2**	AT1G11300	PM	SP	PM
**3**	AT1G11305	-	SP	PM
**4**	AT1G11330	PM	SP	PM
**5**	AT1G11340	PM	SP	PM
**6**	AT1G11350	PM	SP	PM
**7**	AT1G11410	PM	SP	PM
**8**	AT1G34300	PM	SP	PM
**9**	AT1G61360	PM	SP	PM
**10**	AT1G61370	PM	SP	PM
**11**	AT1G61380	PM	SP	PM
**12**	AT1G61390	PM	MT	PM
**13**	AT1G61400	PM	MT	PM
**14**	AT1G61420	PM	SP	PM
**15**	AT1G61430	PM	SP	PM
**16**	AT1G61440	PM	SP	PM
**17**	AT1G61480	PM	SP	PM
**18**	AT1G61490	PM	SP	PM
**19**	AT1G61500	PM	SP	PM
**20**	AT1G61550	PM	SP	PM
**21**	AT1G61610	PM	SP	PM
**22**	AT1G65790	PM	SP	PM
**23**	AT1G65800	PM	SP	PM
**24**	AT1G67520	PM	SP	PM
**25**	AT2G19130	PM	SP	PM
**26**	AT2G41890	PM	SP	PM
**27**	AT3G16030	PM	SP	PM
**28**	AT4G00340	PM	SP	PM
**29**	AT4G03230	PM	SP	PM
**30**	AT4G11900	PM	SP	PM
**31**	AT4G21380	PM	SP	PM
**32**	AT4G21390	PM	SP	PM
**33**	AT4G27290	PM	SP	N / PM
**34**	AT4G27300	PM	SP	PM
**35**	AT4G32300	PM	SP	PM
**36**	AT5G24080	PM	SP	PM
**37**	AT5G35370	PM	SP	PM
**38**	AT5G60900	PM	SP	C / N / PM

^PM, plasma membrane; SP, secretion pathway; MT, mitochondria, C, cytoplasm; N, nucleus.

* “-“ denotes no prediction.
