# Supplementary material for: Classification and phylogenetic analyses of the Arabidopsis and tomato G-type lectin receptor kinases
Source: BMC Genomics. 2018 Apr 6;19:239. doi: 10.1186/s12864-018-4606-0 (PMC5889549; doi:10.1186/s12864-018-4606-0)
Supplement: Supplementary file 9 — Alignment of predicted amino acid sequences of tomato G-LecRKs kinase domains with Solyc03g006720 using ClustalW. Lines on top of alignment show subdomains I and II, ATP binding site (GxGxxGxV) and subdomain VI, the serine/threonine kinase active site (HRDLKxxN). (PDF 7110 kb) [file 12864_2018_4606_MOESM9_ESM.pdf]

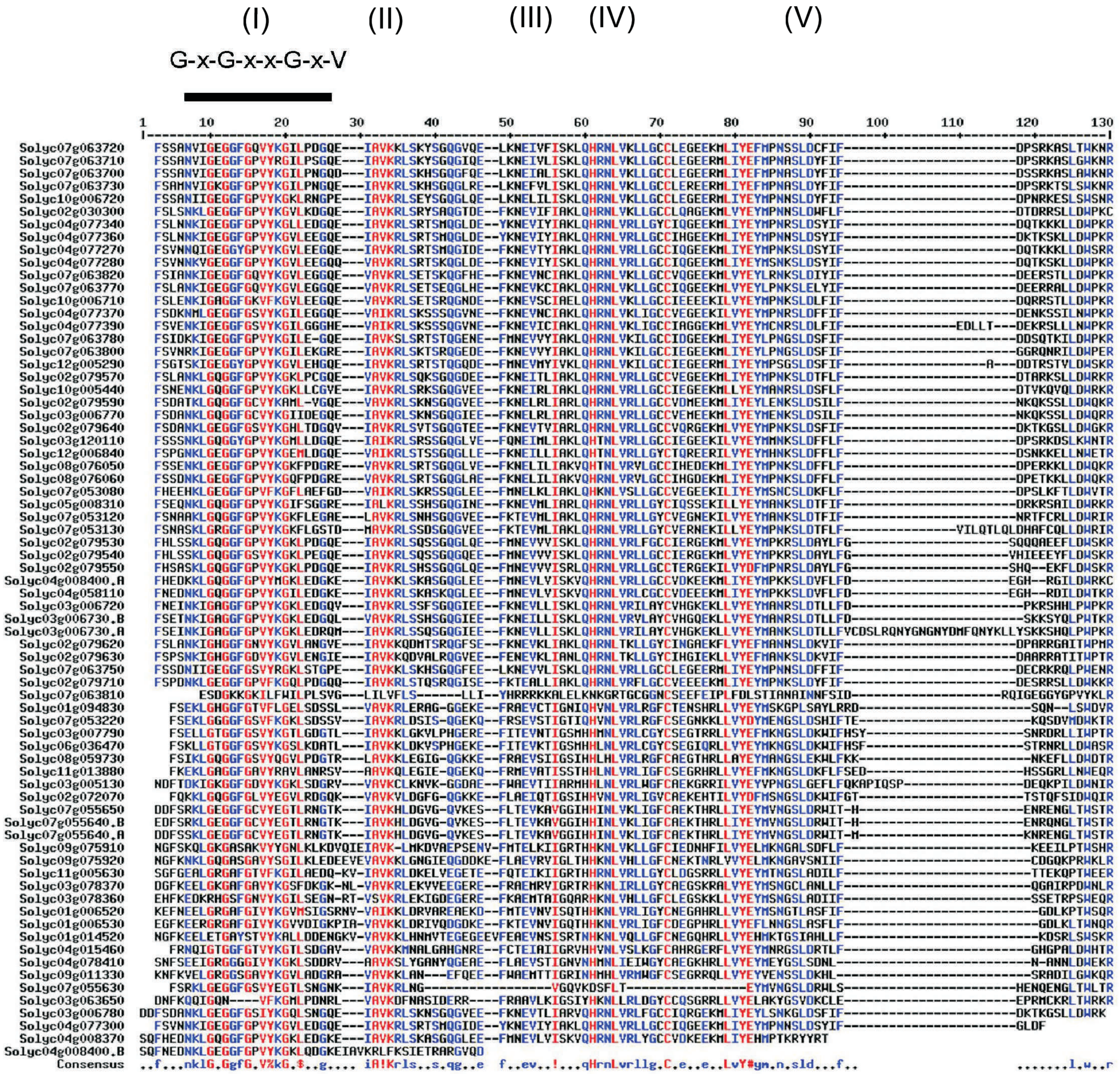
(I)
(II)
(III)
(IV)
(V)
G-x-G-x-x-G-x-V
Consensus

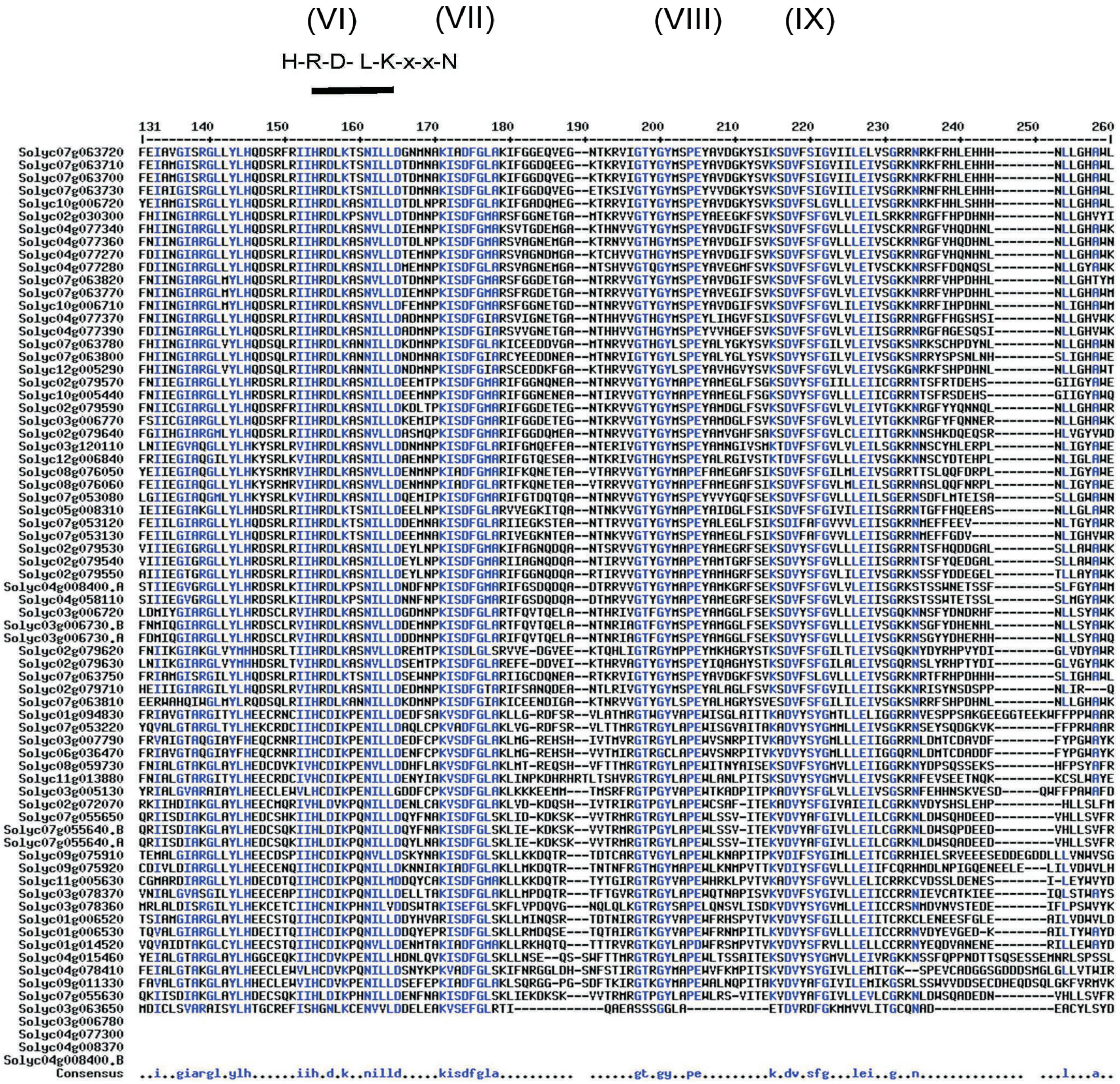

(VI)
(VII)
(VIII)
(IX)
H-R-D- L-K-x-x-N
131 140 150 160 170 180 190 200 210 220 230 240 250 260
Solyc07g063720
Solyc07g063710
Solyc07g063700
Solyc07g063730
Solyc10g006720
Solyc02g030300
Solyc04g077340
Solyc04g077360
Solyc04g077270
Solyc04g077280
Solyc07g063820
Solyc07g063770
Solyc10g006710
Solyc04g077370
Solyc04g077390
Solyc07g063780
Solyc07g063800
Solyc12g005290
Solyc02g079570
Solyc10g005440
Solyc02g079590
Solyc03g006770
Solyc02g079640
Solyc03g120110
Solyc12g006840
Solyc08g076050
Solyc08g076060
Solyc07g053080
Solyc05g008310
Solyc07g053120
Solyc07g053130
Solyc02g079530
Solyc02g079540
Solyc02g079550
Solyc04g008400.A
Solyc04g058110
Solyc03g006720
Solyc03g006730.B
Solyc03g006730.A
Solyc02g079620
Solyc02g079630
Solyc07g063750
Solyc02g079710
Solyc07g063810
Solyc01g094830
Solyc07g053220
Solyc03g007790
Solyc06g036470
Solyc08g059730
Solyc11g013880
Solyc03g005130
Solyc02g072070
Solyc07g055650
Solyc07g055640.B
Solyc07g055640.A
Solyc09g075910
Solyc09g075920
Solyc11g005630
Solyc03g078370
Solyc03g078360
Solyc01g006520
Solyc01g006530
Solyc01g014520
Solyc04g015460
Solyc04g078410
Solyc09g011330
Solyc07g055630
Solyc03g063650
Solyc03g006780
Solyc04g077300
Solyc04g008370
Solyc04g008400.B
Consensus
..i..giargl.ylh......iih.d.k..nilld.....kisdfgla..........
......gt.gy..pe.........k.dv.sfg...lei..g..n..............
...l...a..

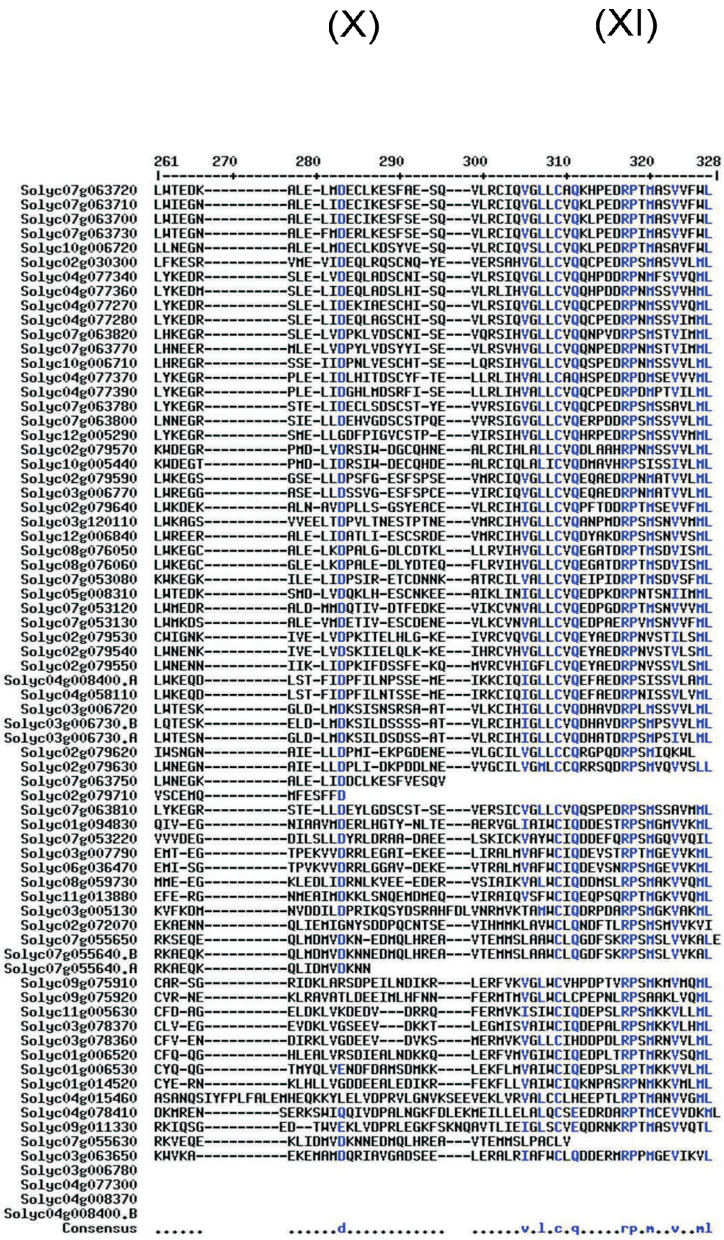
(X)
(XI)
