# Supplementary material for: Classification and phylogenetic analyses of the Arabidopsis and tomato G-type lectin receptor kinases
Source: BMC Genomics. 2018 Apr 6;19:239. doi: 10.1186/s12864-018-4606-0 (PMC5889549; doi:10.1186/s12864-018-4606-0)
Supplement: Supplementary file 12 — Subcellular localization of the tomato G-LecRKs. Localization was predicted using TargetP and CELLO software programs/tools. (PDF 56 kb) [file 12864_2018_4606_MOESM12_ESM.pdf]

Additional file 12. Subcellular localization of the tomato G-LecRKs. Localization was predicted using TargetP and CELLO software programs/tools.

	Gene	TargetP	CELLO
**1**	Solyc01g006520	SP^	PM
**2**	Solyc01g006530	SP	PM / N
**3**	Solyc01g094830	SP	PM
**4**	Solyc02g030300	-*	PM
**5**	Solyc02g072070	SP	PM
**6**	Solyc02g079530	SP	PM
**7**	Solyc02g079540	SP	PM
**8**	Solyc02g079550	SP	PM
**9**	Solyc02g079570	SP	PM
**10**	Solyc02g079590	SP	PM
**11**	Solyc02g079620	SP	PM
**12**	Solyc02g079630	CH	EX / PM
**13**	Solyc02g079640	SP	PM
**14**	Solyc02g079710	SP	PM
**15**	Solyc03g005130	SP	PM
**16**	Solyc03g006720	SP	PM
**17**	Solyc03g006730.A	SP	PM
**18**	Solyc03g006730.B	MT	PM
**19**	Solyc03g006770	SP	PM
**20**	Solyc03g006780	SP	PM
**21**	Solyc03g007790	SP	PM
**22**	Solyc03g063650	SP	PM
**23**	Solyc03g078360	SP	PM
**24**	Solyc03g078370	SP	PM
**25**	Solyc03g120110	SP	PM
**26**	Solyc04g008370	SP	PM / N
**27**	Solyc04g008400.A	SP	PM
**28**	Solyc04g008400.B	-	PM / N
**29**	Solyc04g015460	SP	PM
**30**	Solyc04g058110	SP	PM
**31**	Solyc04g077270	SP	PM
**32**	Solyc04g077280	SP	PM
**33**	Solyc04g077300	SP	PM
**34**	Solyc04g077340	SP	PM
**35**	Solyc04g077360	SP	PM
**36**	Solyc04g077370	SP	PM
**37**	Solyc04g077380	-	EX
**38**	Solyc04g077390	SP	PM

**39**	Solyc04g078410	SP	PM
**40**	Solyc05g008310	SP	PM
**41**	Solyc06g036470	SP	EX / PM
**42**	Solyc07g053080	SP	PM
**43**	Solyc07g053120	SP	PM
**44**	Solyc07g053130	SP	PM
**45**	Solyc07g053220	SP	PM
**46**	Solyc07g055630	-	N / C / MT
**47**	Solyc07g055640.A	MT	MT
**48**	Solyc07g055640.B	-	C / N
**49**	Solyc07g055650	-	PM / MT
**50**	Solyc07g063700	SP	PM
**51**	Solyc07g063710	SP	PM
**52**	Solyc07g063720	SP	PM
**53**	Solyc07g063730	SP	PM
**54**	Solyc07g063750	SP	PM
**55**	Solyc07g063770	SP	PM
**56**	Solyc07g063780	SP	PM
**57**	Solyc07g063800	SP	PM
**58**	Solyc07g063810	MT	PM
**59**	Solyc07g063820	SP	PM
**60**	Solyc08g059730	SP	PM
**61**	Solyc08g076050	SP	PM
**62**	Solyc08g076060	-	PM
**63**	Solyc08g076070	-	N
**64**	Solyc09g011330	SP	PM
**65**	Solyc09g075910	SP	PM
**66**	Solyc09g075920	SP	PM
**67**	Solyc10g005440	SP	PM
**68**	Solyc10g006710	SP	PM
**69**	Solyc10g006720	SP	PM
**70**	Solyc11g005630	MT	PM
**71**	Solyc11g013880	SP	PM
**72**	Solyc12g005290	SP	PM
**73**	Solyc12g006840	SP	PM

^PM, plasma membrane; SP, secretion pathway; MT, mitochondria, C, cytoplasm; N, nucleus; CH, chloroplast; EX, extracellular.

* “-“ denotes no prediction.
